# Physical and 3D numerical modelling of reinforcements pullout test

**DOI:** 10.1038/s41598-024-57893-3

**Published:** 2024-03-28

**Authors:** Ivan P. Damians, Aníbal Moncada, Sebastià Olivella, Antonio Lloret, Alejandro Josa

**Affiliations:** 1 Department of Civil and Environmental Engineering (DECA), Universitat Politècnica de Catalunya·BarcelonaTech (UPC), Barcelona, Spain; 2 International Centre for Numerical Methods in Engineering (CIMNE), Barcelona, Spain; 3 VSL International Ltd, Barcelona, Spain

**Keywords:** 3D modelling, Soil-reinforcement interaction, Pullout tests, Finite element modelling, Reinforced soil walls, Polymeric strip reinforcement, Steel ladder reinforcement, Solid Earth sciences, Engineering, Mathematics and computing

## Abstract

This paper reports results of laboratory and 3D numerical modeled pull-out tests with steel ladders and polymeric strip reinforcements. These types of reinforcement are commonly used in reinforced soil walls constructed with concrete facing elements. Laboratory pull-out tests are required to determine accurate and realistic pull-out strength values considering the interaction of specific reinforcement and backfill materials under different confining pressures (i.e., trying to simulate the different reinforcement layer arrangements and load conditions in actual reinforced soil walls). International design Codes for reinforced soil walls provide default values for pull-out strength. However, in many cases, default values are too conservative and/or are not strictly specified for particular reinforcement types. Pull-out tests can be difficult and expensive to perform, thus not being common nor worth for the vast majority of reinforced soil wall projects. Consequently, calibrated numerical models can be useful to predict pull-out response under site-specific conditions, and provide further understanding of the mechanisms involved in the soil-reinforcement interaction. Details of the numerical approach, including relevant aspects of the soil-reinforcement interfaces, are described. Examples of calibrated numerical predictions for pull-out loads, displacements, and soil-dilatancy effects are presented. The influence of reinforcement, soil and interface stiffnesses is shown. Numerical results provide useful insight for future modelling works of the complex interaction between type-specific backfill materials and reinforcement element, relevant for investigation and/or practical design of reinforced soil walls.

## Introduction

Design of reinforced soil walls (RSWs) typically considers working stress conditions (i.e., far away from failure), meaning strains are not enough to fully develop the soil-reinforcement interface strength, even for extensible reinforcements, in which maximum strains are not expected to surpass a 1% threshold^1^. Accurate designing of RSWs requires a proper characterization of the interface shear behaviour between the embedded reinforcement elements and surrounding soil material. Pullout tests are particularly useful to study the shear response between soil and reinforcement materials, allowing to quantify interface strength and stiffness parameters required for an optimized reinforcement design while ensuring safety conditions.

Ample research of pullout test data for steel and polymeric reinforcements with varied geometries (i.e., ladders, grids, mats, strips, among others) is available in the literature. Pullout failure can be troublesome depending on the reinforcement geometry and surcharge conditions^2^ as well as backfill material characteristics^3–5^. The pullout response between metallic and polymeric reinforcement has proven to be drastically different^6^. Metallic (i.e. inextensible) reinforcement presents an instantaneous stress–strain response throughout the material, while in polymeric (i.e., extensible) reinforcement, the stress-stress response is gradual and varies from the head to rear. Latest research still includes experimental work (e.g., Gergiou et al.^7^), as well as a special focus on model accuracy and reliability assessments of current design methods (e.g.,^8–12^), where it has been stated that the pullout limit state can have practical variations depending on the chosen load model.

Numerical methods have been used to replicate the pullout behaviour in reinforced soil structures, either by discrete element (e.g.,^13,14^) or finite element (e.g.,^15,16^) methods. Reported results have shownproper adjustments between simulated and measured values, providing evidence of the accuracy of numerical tools.

The present study focuses, first, in laboratory measured data from pullout tests using steel ladder and polymeric strip reinforcements following the requirements of ASTM D6706-01^17^ and EN 13738^18^ and lessons learned from previous cases in the literature (e.g.,^19,20^, among others). Measured results were compared with past measured and theorical data available in the literature as well as international codes. Second, a 3D finite element model was implemented to simulate and analyze pullout response. A base case was defined with typical properties considering frequently used backfill soils in RSWs. Sensitivity analyses were carried out over model parameters, followed by a calibration process which took into account the measured steel ladder and polymeric strip pullout test data.

## Pullout resistance

The stress-transfer mechanism of soil-reinforcement pullout interactions depend on reinforcement type and configuration, soil properties, and applied stress. For strips and sheet reinforcement, the pullout resistance is equal to the frictional shear stresses over the whole contact area between soil and reinforcement. For bar-mat, ladder, grid, or ribbed strip reinforcements, a complementary passive strength or bearing resistance is developed due to the transversal member surfaces, in which dilatancy, reinforcement roughness and soil stress state come into play^21^. The pullout resistance (P_r_) can be expressed as follows (Eq. 1):

1 $${{\text{P}}}_{{\text{r}}}={{\text{f}}}^{\mathrm{^{\prime}}}\left({{\text{CwL}}}_{{\text{e}}}^{\mathrm{^{\prime}}}{\upsigma }_{{\text{v}}}\right)$$

2 $${{\text{f}}}^{\mathrm{^{\prime}}}=\mathrm{\alpha }{{\text{F}}}^{*} =\mathrm{\alpha }{{\text{R}}}_{{\text{i}}}\mathrm{ tan }(\upphi )$$

Here, f’ is the friction interaction factor between the soil and the reinforcement, C is the overall reinforcement surface area geometry factor (i.e., equal to 2 for strips and ladder as in two contact faced-reinforcement configuration systems), w is the width of the reinforcement, and ${{\text{L}}}_{{\text{e}}}^{\mathrm{^{\prime}}}$ is the effective reinforcement length in the resisting zone.

The friction interaction factor f^’^ will vary depending on the refenced code. In the case of AASHTO^22^, a scale effect correction factor (α), and a pullout friction factor (F^*^) are proposed. Factor α is assume to be 1 for inextensible reinforcement, and less than 1 for extensible reinforcements. Factor F^*^ is a reduction of soil strength via an interaction coefficient, R_i_, and the soil friction angle, ϕ (Eq. 2). For geosynthetic materials (i.e., geogrids, geotextiles, and geostrips), R_i_ has a proposed value of 0.67^22^. In the case of polymeric strips, values of R_i_ = 0.8 can be conservatively assumed in the absence of test data^23,24^. By means of statistical analysis, Miyata et al.^12^ showed that the accuracy of linear pullout models for polymeric strips will depend on the magnitude of predicted pullout capacity and vertical stress acting over the reinforcement, which is generally not desired in design methodologies. For bar-mat or steel ladders, the value of F* can be obtained as a relationship between thickness of the transversal bar members, t, and separation between transversal bar members, S_t_, as follows (Eq. 3):

3 $${{\text{F}}}^{*}={{\text{n}}}_{{\text{q}}}\left({\text{t}}/{{\text{S}}}_{{\text{t}}}\right)$$

Here, n_q_ is a bearing capacity factor that varies linearly with depth from n_q_ = 20 at surface level (z = 0) to n_q_ = 10 at depths of 6 m or more. Values of n_q_ mean F* will be a linearly decreasing function from 0 to 6 m of depth, and constant for greater depths. Pullout models based on grid geometry and containing empirical parameters have shown to perform better than purely theoretical bearing capacity and soil friction angle models^1,25^

In the case of NF P 94-270^26^, f´ is related to an apparent soil-reinforcement interaction coefficient, μ^*^_(z)_, which varies with soil gradation, transversal bar diameter and separation for steel ladders, and soil gradation and soil friction angle ϕ for polymeric strips. As with F^*^ (from AASHTO^22^, the value of μ^*^_(z)_ decreases linearly until 6 m of depth, after which it remains constant.

**Figure 1:**
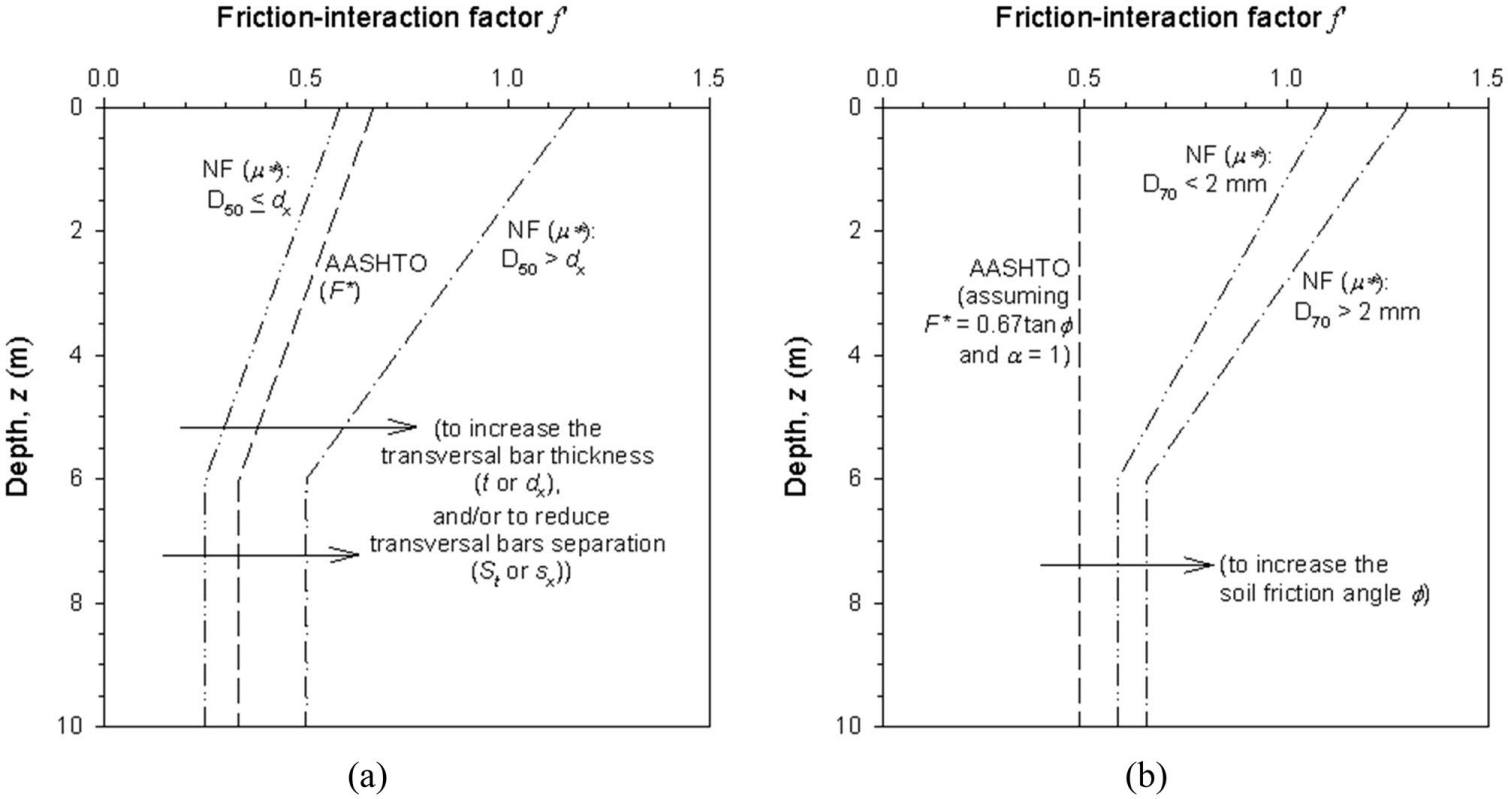
Friction-interaction factor (f^’^) according to AASHTO^22^ and NF^26^ codes adapted to (**a**) steel strips and (**b**) polymeric reinforcements under backfill soil types 1 (draining) and 2 (granular). In steel ladder case transversal bar thickness and separation assumed as 10 and 300 mm, respectively; backfill friction angle assumed as 36° with soil Cu > 2 for polymeric strip case.

Figure 1 compares the values of f´ obtained through AASHTO^22^ and NF^26^ guidelines. For steel ladder reinforcements (Fig. 1a), a transversal bar separation of 300 mm with 10 mm-diameter bars is assumed. For polymeric strips (Fig. 1b) a soil friction angle ϕ  = 36° and a coefficient of uniformity C_u_ > 2 is assumed. Clear variations between design codes evidence the need for laboratory pullout tests to obtain valuable data concerning the combined response of project specific type of reinforcement, loading conditions, and fill material characteristics.

If required, using on Eq. (1) and AASHTO^22^ guidelines, a simple modification can be carried out to incorporate cohesion in the pullout resistance using a frictional (f^’^) and cohesion (f_c_^’^) friction interaction factors, as follows (Eq. 4):

4 $${{\text{P}}}_{{\text{r}}}={{\text{f}}}^{\mathrm{^{\prime}}}\left(2{{\text{wL}}}_{{\text{e}}}^{\mathrm{^{\prime}}}{\upsigma }_{{\text{v}}}\right)+{{{\text{f}}}_{{\text{c}}}}^{\mathrm{^{\prime}}}\left(2{{\text{wL}}}_{{\text{e}}}^{\mathrm{^{\prime}}}{\upsigma }_{{\text{v}}}\right)=\left({{\text{F}}}^{*}\mathrm{\alpha }\right)2{{\text{wL}}}_{{\text{e}}}^{\mathrm{^{\prime}}}{\upsigma }_{{\text{v}}}+\left({{\text{c}}}_{{\text{i}}}\mathrm{\alpha }\right)2{{\text{wL}}}_{{\text{e}}}^{\mathrm{^{\prime}}}{\upsigma }_{{\text{v}}}$$

Here, c_i_ is the soil-reinforcement interface cohesion, understood as soil-reinforcement adherence, reduced from the fill-soil cohesion using the frictional interaction coefficient (i.e., R_i_).

## Model test

### Test apparatus and methodology

**Figure 2:**
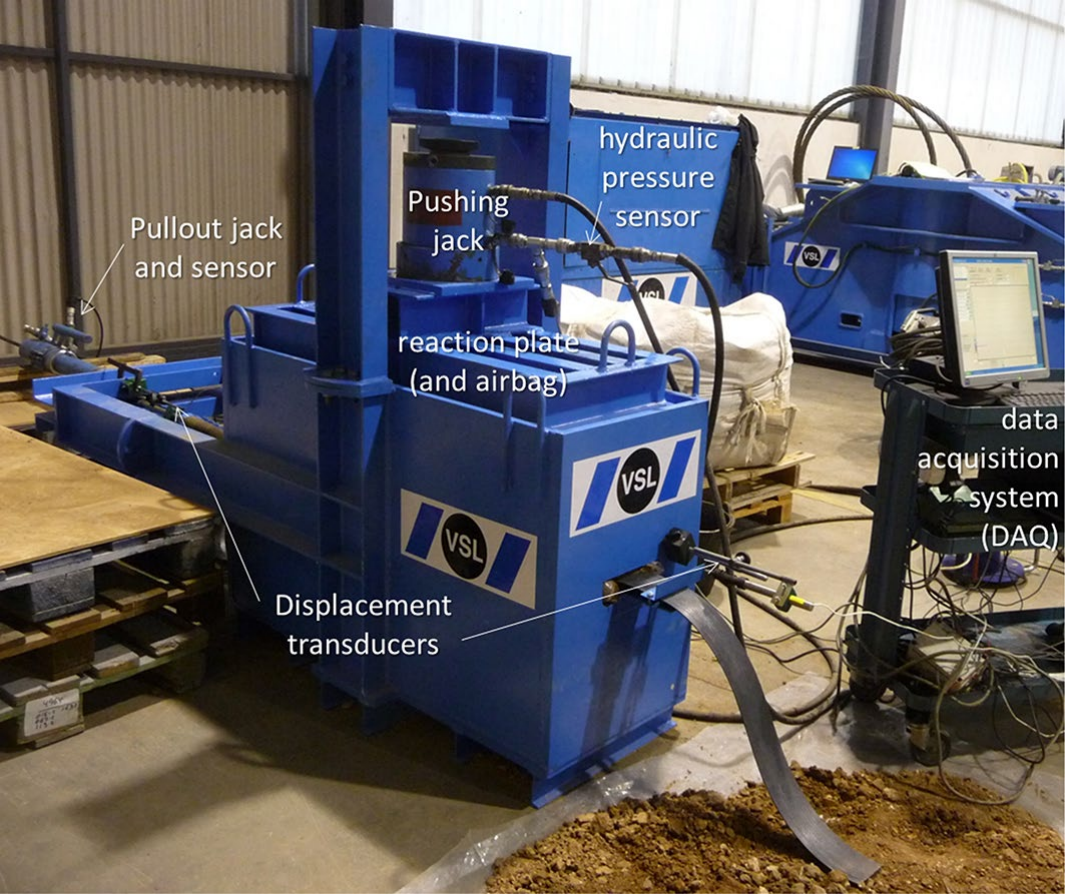
Pullout test box setup and main components.

Test were carried out using a rigid steel box with an upper opening for material manipulation and to apply vertical loads (see Fig. 2). The pullout box dimensions are 1250 mm-length, 500 mm-width, and 550–750 mm-high. The chosen dimensions allow for minimum embedment length, minimum top and bottom soil depth, and enough distance to the lateral boundaries, as recommended by ASTM D6706-1^17^ and BS EN 13738^18^. The front side includes an opening and metal sleeve through which reinforcements are connected to clamps and pulling mechanism. The sleeve opening is 200 mm-wide and 40 mm-high, and goes 250 mm into the pullout box to avoid the influence of boundary conditions. The rear side includes openings to measure relative displacements or fix the end of the reinforcements as needed. All openings are located at the middle of the box height, in the same horizontal level. The test box includes two out-front arms over which the pulling jack sits and acts as a reaction to the pullout force.

Prior to filling the test box, all sides were cleaned, lubricated, and covered with plastic films to reduce lateral friction effects and fill adhesion. Soil was placed uniformly in layers of approximately 150 mm and compacted using a plate compactor. Unit weight of ≥ 95% of the modified proctor test results was ensured by means of densitometer measurements. After each pullout tests all of the soil above the reinforcement specimen, plus 5–10 cm of soil below the reinforcement specimen layer was carefully removed and replaced.

Vertical loads were applied using a pushing jack device over a loading plate, achieving proper stress distributions by placing a pneumatic bag over the compacted soil. Loads scenarios were include equivalent depths ranging from 0.370 to 10 m (considering considered the overburden pressure and the self-weight of the fill material). Vertical loads were measured constantly throughout each test.

Pullout tests were carried out with an axial constant rate of displacement of 1 mm/min using a calibrated jack pump. Reinforcements were connected using clamps that prevent any slipping or relative displacements and allow for a uniform load transmission. A metal sleeve in the frontal opening ensures no load transfer to the reinforcement at the boundary zone.

Displacements were measured at the front and back of reinforcements using transducer devices with 0.01 mm-accuracy. Measurements were taken every 0.2 mm of displacements or 6 s intervals of time. If no failure occurred, tests were carried out until displacements of 20 mm for steel ladders, and 15 mm polymeric strips (measured at the tail of the specimen).

### Test materials

Tested reinforcements included steel ladders and polymeric strips. Ladders had widths of 160–170 mm with 8 or 12 mm-thick transversal bars, spaced 150- or 300-mm. Strips are composed of high tenacity polyester (PET) fibers bundled in a polyethylene sheath. PET strips with an ultimate tensile strength of 30 and 70 kN were tested using a linear configuration (i.e., parallel to the pullout direction). In all cases, a minimum separation of 100 mm from the reinforcement and the sidewalls was ensured.

As per AASHTO﻿^22^, soil gradation shall not be gap-graded and satisfy a well-graded classification based on ASTM D2487^27^, meaning, a coefficient of uniformity (C_u_ = D_60_/D_10_) greater than 6 for a sandy soil (SW) and greater than 4 for a well graded gravel (GW). The coefficient of curvature (C_c_ = (D_30_^2^)/(D_60_ D_10_)) must take values from 1 to 3. Plasticity index (PI) must be equal or below 6. Proper fill-soil can be classified as draining (Type 1), granular (Type 2), and intermediate (Type 3), depending on gradation and plasticity index^28^. The use of Type 3 soil as fill material is subjected to specific studies regarding the structure conditions.

36 tests were carried out with steel ladder reinforcements, including 3 ladders configurations and 12 different soils (i.e., 12 series of tests with different confinement pressures). All soils consisted of granular fills with friction angles ϕ from 31° to 40°, unit weight γ from 18.8 to 22.5 kN/m^3^ and coefficient of uniformity C_u_ > 2.

For PET strip reinforcements, confinement pressures simulated 1-, 3.5-, and 7-m of depth. The fill soil classified as low plasticity silty sand (SM(L)) with unit weight, γ = 21 kN/m^3^, friction angle, ϕ = 31.4°, cohesion, c = 14 kPa, diameter D_50_ of 3–4 mm, coefficient of uniformity, C_u_ = 700, coefficient of curvature, C_c_ = 0.275, and plasticity index, PI = 6.6. Friction angle and cohesion values for all soil materials were obtained from direct shear tests. This soil does not satisfy the plasticity and gradation code recommendations and falls into a Type 3 fill as per EN 14475^28^, meaning that it is only suitable for use if pullout tests results show an adequate performance.

### Test results

#### Steel ladder pullout tests

**Figure 3:**
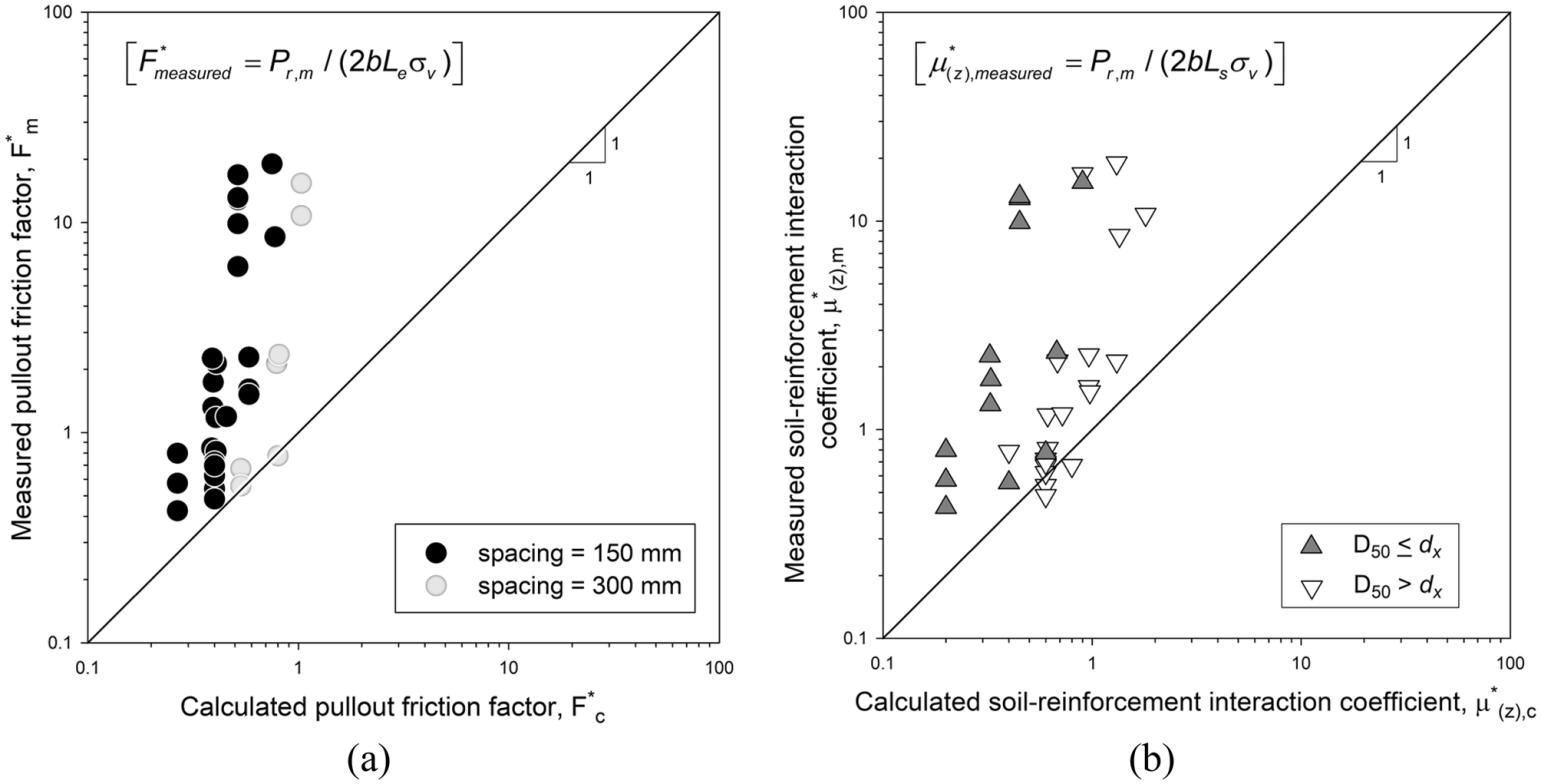
(**a**) AASHTO- and (**b**) NF-calculated versus measured friction interaction factor (f’) for pullout tests with steel ladders.

Figure 3 shows the ratio between calculated (be it AASHTO or NF) and measured friction interaction factor for a wide range of confinement pressures. Values greater than one reflect conservative results, while lower than unit values correspond to a lack of safety. Overconservative results were observed at low confinement pressures for AASHTO (Fig. 3a) and NF-calculated (Fig. 3b) values. Values tend towards the unit at increase depths or higher confinement pressures (i.e., lower f´ values). The ratio between measured and NF-calculated values tended to higher values when D_50_ ≤ d_x_ (i.e., the sieve passing 50% of the soil mass (D_50_) is lower than the transversal bar thickness), (i.e., more conservative) compared to the D_50_ > d_x_ results.

**Figure 4:**
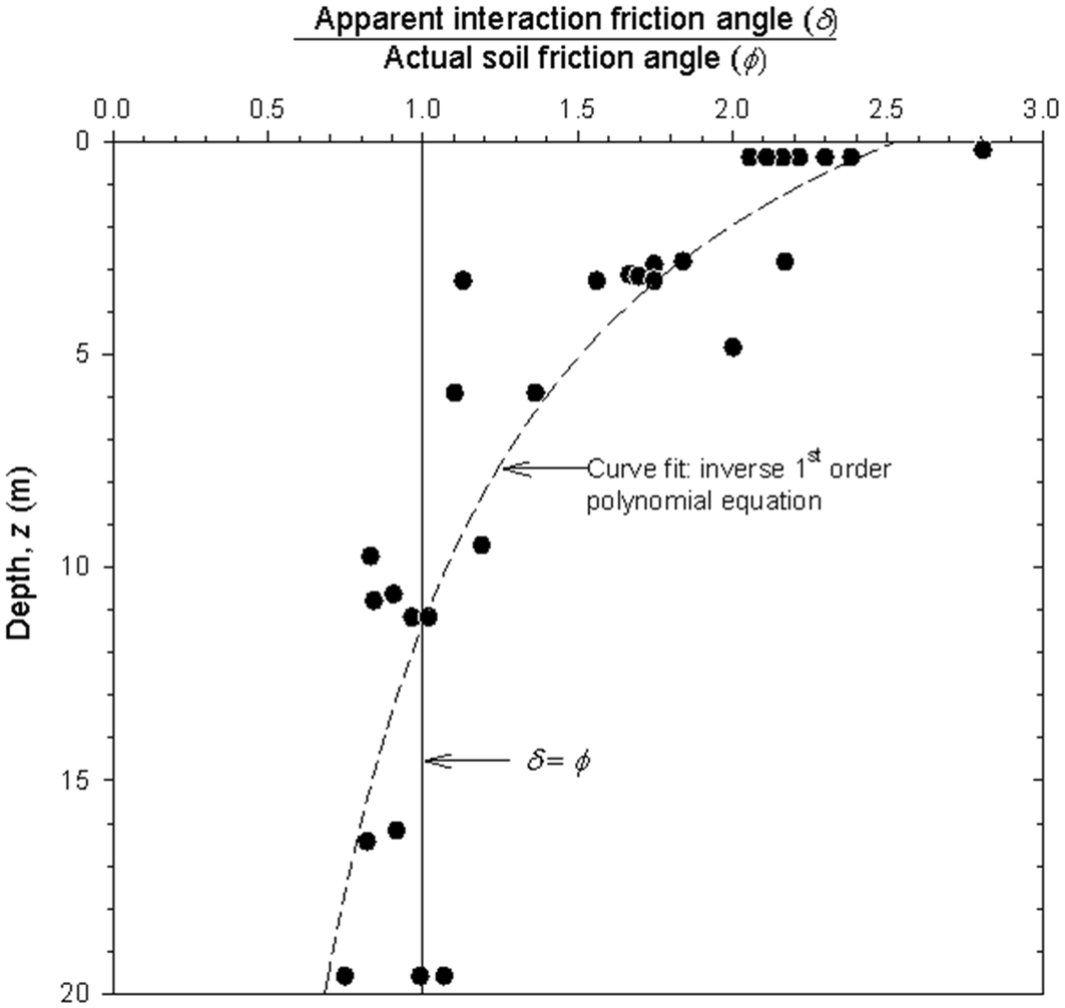
Ratio between apparent pullout interaction friction angle (δ) and actual soil friction angle (ϕ) for steel ladder reinforcement pullout tests.

Figure 4 shows the ratio between apparent soil-reinforcement interaction friction angle (i.e., δ = tan^−1^(f`)) and the actual soil friction angle (ϕ). High values of δ were measured at low confining pressures (as high as δ > 80°), which agrees with results reported by Ingold^29^. Increased δ can be understand as a product of soil dilation. As confining pressure increases, the ratio between angles decreases. δ values below 1 (i.e., δ <  ϕ) under 10 m of depth could imply that not only bearing strength capacity is developed in steel ladders-soil interaction (representative in cases with δ ≥ ϕ), but also frictional (where typically δ < ϕ values are reached, being the soil friction angle the higher boundary of δ values).

**Figure 5:**
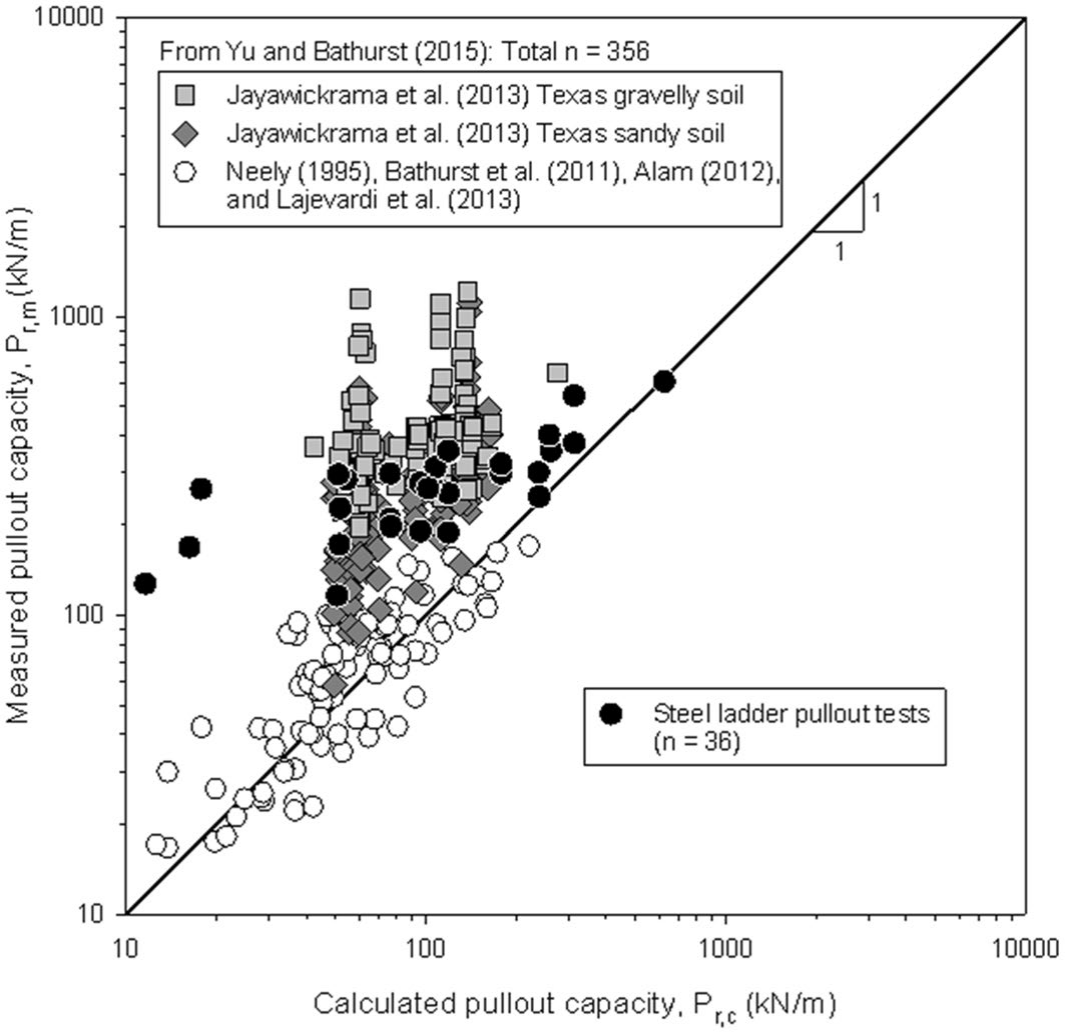
Measured and calculated pullout capacity according to AASHTO^22^ for steel ladder reinforcement pullout tests and other bar-mat reported cases (adapted from^30^).

Figure 5 compares measured and AASHTO-calculated pullout strength capacity (P_r_) values obtained in the present study and those collected by Yu and Bathurst^30^. As previously stated, for low confining pressures, the overestimation of the calculated pullout capacity is considerable. Results from this study are coherent with those obtained by Jayawickrama et al.^31^ at similar confinement pressures.

#### Polymeric strip pullout tests

**Figure 6:**
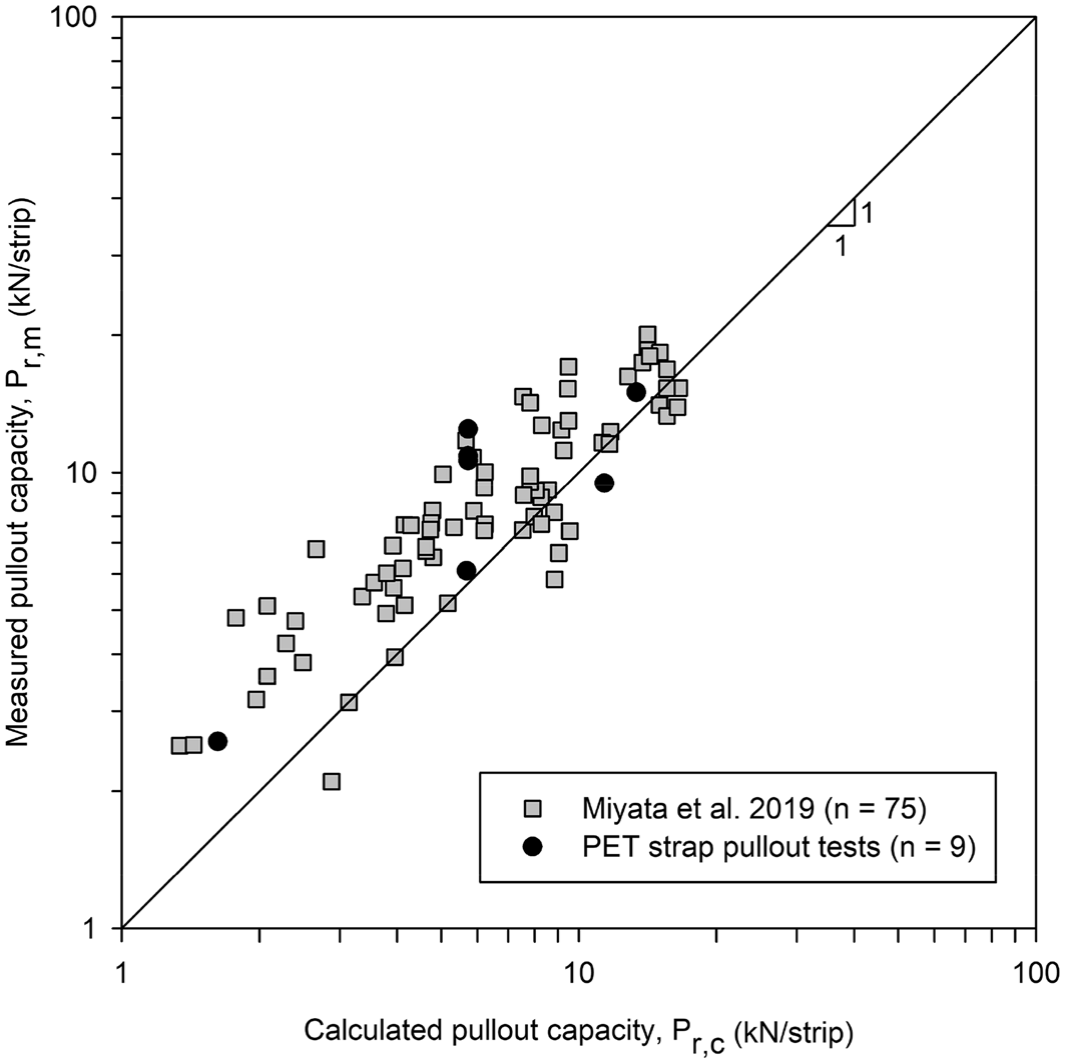
Calculated and measured values of pullout capacity according to AASHTO^22^ for single polymeric strip reinforcements (adapted from^12^).

Figure 6 shows the comparison of measured and AASHTO-calculated pullout strength capacity (P_c_) for single polymeric (PET) strip reinforcements. Values are compared to the extensive data compiled and reported by Miyata et al.^12^. Calculated values consider conservative-default F’ = 0.67tan(ϕ) and α = 1.0. Results from Miyata et al.^12^ show that, on average, calculated values are conservative. Nevertheless, there are cases in which the calculated pullout strength was overestimated which appear to occur with more frequency at higher confining pressures (i.e., greater depths). Results obtained in the present study, while scarce, appear to follow the same trend as those presented in Miyata et al.^12^.

## 3D finite element numerical model

### Model description

**Figure 7:**
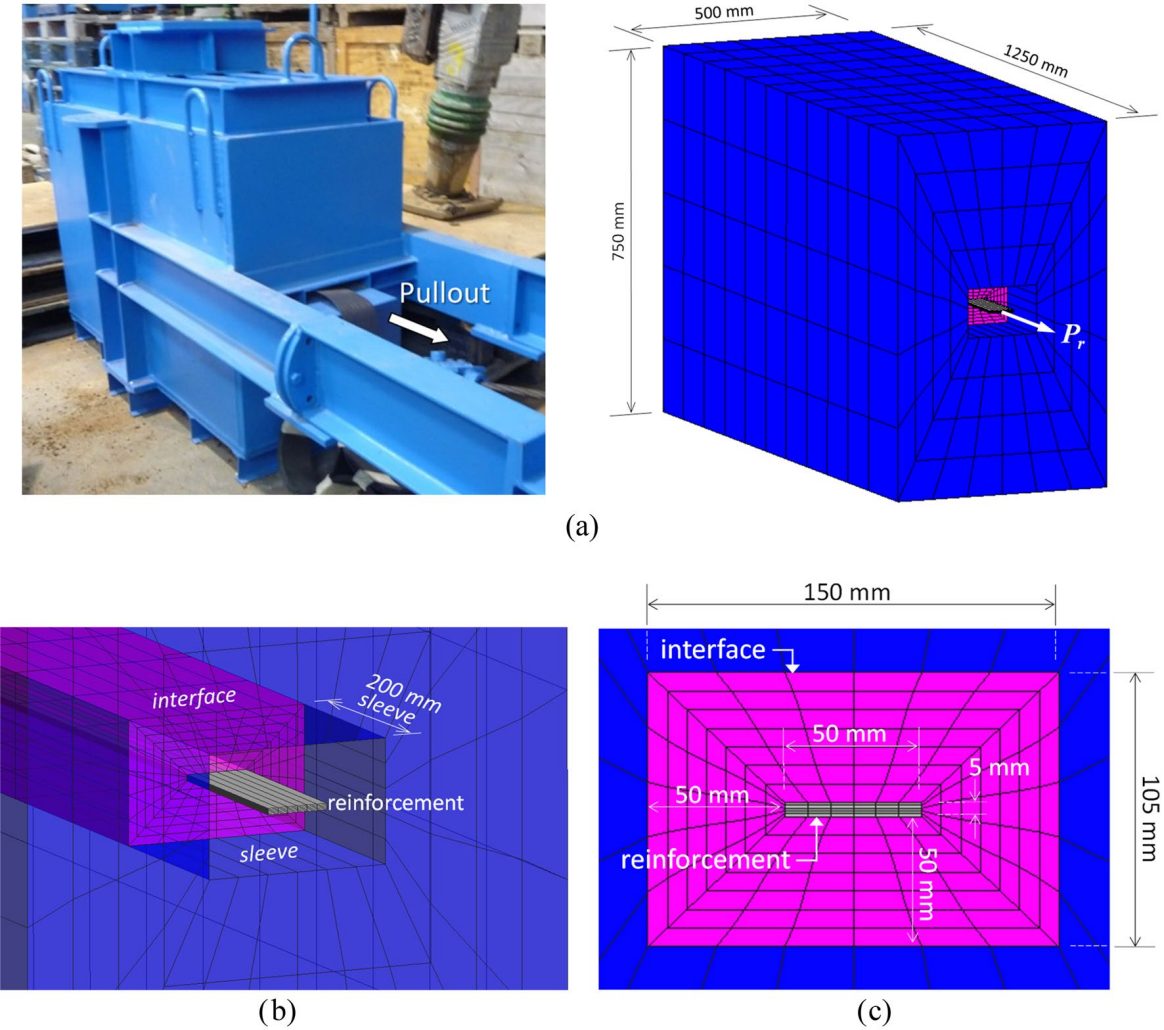
Pullout 3D numerical model: (**a**) pullout box/domain geometry, (**b**) frontal box opening sleeve, and (**c**) soil-reinforcement interface mesh detail.

A 3D model was implemented to simulate pullout test using the finite element software CODE_BRIGHT^32^. Figure 7a shows the model domain, which replicates the test box dimensions. As with the test box, the model geometry includes a frontal opening with a 200 mm-length sleeve (Fig. 7b). 3D numerical models can provide accurate stress–strain simulations at the expense of increased computational cost and can be of use to evaluate performance of 2D models.

Metallic and polymeric reinforcements were modeled via an equivalent strip with dimension 50 mm-width, 5 mm-thick, and 1050 mm-length (Fig. 7c), and a linear elastic constitutive law. The soil-reinforcement interface was implemented using continuum elements. Numerical results regarding load transfers between materials using continuum element interfaces have shown good agreement when compared to zero-thickness interface elements available in other numerical software and allow for more control over strength and stiffness variations between materials as well as element shapes and sizes^33,34^ and have been previously used in 3D numerical modelling of reinforced soil walls^35^. Soil fill and interface materials were modeled using a linear elastic stiffness with a Mohr–Coulomb plastic law with dilatancy.

**Figure 8:**
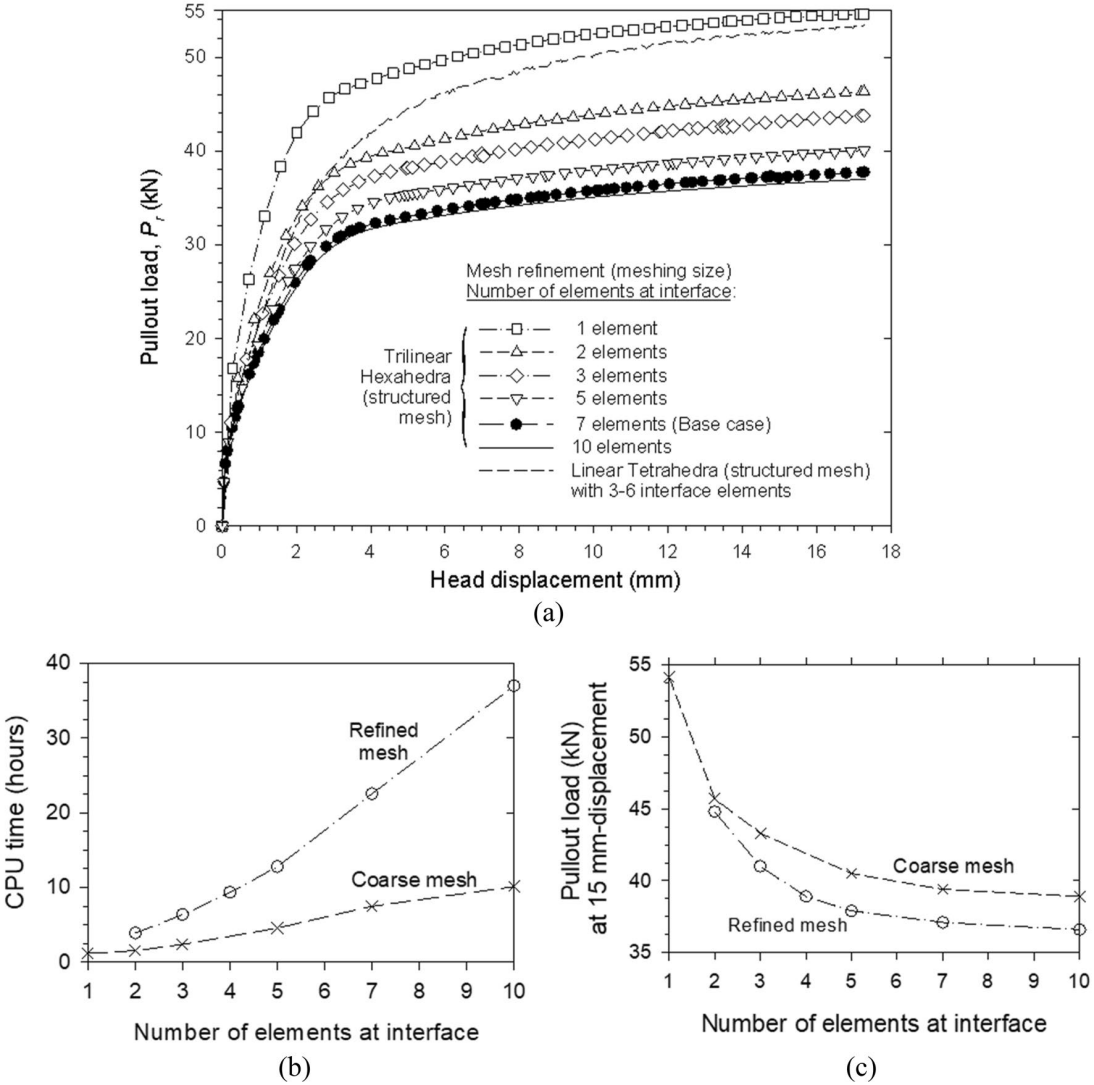
(**a**) Reinforcement pullout load versus axial displacement with regards to interface finite element mesh refinement and (**b**) required CPU time and (**c**) maximum pullout load with regard to interface mesh refinement.

A a-prior mesh optimization process was undergone. Figure 8a shows the pullout load–displacement results with regards to the interface mesh refinement. As the number of elements in the interface increase, modelled pullout capacity is reduced by approximately 35% (i.e., from 54 to 37 kN), reaching an asymptotic value with a 10-element interface. As expected, the number of interface elements increases computation time considerably (Fig. 8b). A refined mesh yielded lower (deemed more accurate) pullout loads (Fig. 8c). Different mesh arrangements of the fill soil, in addition to the number of interface elements, yielded similar trends. A 7-element interface with structured trilinear hexahedrons (i.e., brick elements) was deemed as the optimal mesh geometry.

The pullout test procedure was replicated using three stages, consisting of 12 steps. For stage one, an equilibrium state is calculated from steps 0 through 10. For stage two, the confining pressure for each scenario is applied using a vertical surcharge on top of the box as a ramp load from step 10 to 11. During stage three, from step 11 to 12, the pullout load is applied as a constant velocity-displacement of 2 × 10^–6^ m/s at the front of the reinforcement strip, which generates 17.28 cm of pullout displacement by the end of the simulation.

### Base case

The initial (i.e., base) case consisted of a steel strip reinforcement and regular granular fill soil. Table 1 details material properties for the base case. Values were selected to be in agreement with the reinforced backfill material reported by Runser^36^. An equivalent depth of z = 3 m was achieved with a vertical surcharge of 52.5 kPa, which, in addition to the 0.375 m of soil fill above the reinforcement, results in a theorical vertical surcharge of 60 kPa.

**Table 1:** Pullout model base case material properties.

Parameters	Materials		
	Reinforcement	Fill soil	Interface
Unit weight, γ_n_ (kN/m^3^)	75	20	20
Elastic stiffness modulus, E (MPa)	210 000	20	20
Poisson’s ratio, ν (–)	0.3	0.3	0.45^a^
Cohesion, c (kPa)	–	1	1
Friction angle, ϕ and δ (°)	–	ϕ_s_ = 38	δ = 28.7^b^
Dilatancy angle, ψ (°)	–	8 ^c^	8^d^

^a^Despite demonstrated to have no much significant effect on pullout capacity, higher ν-value (i.e., ν_i_ = 0.45) were considered to best fit pullout capacity under high vertical pressure cases to mobilize confinement (i.e., to increase mean stress p-invariant) and reduce unrealistic volumetric interface plasticization.

^b^δ = 28.8° (= ϕ_i_) is equivalent to an interface reduction factor of R_i_ = tanδ/tanϕ = 0.7, which corresponds to an AASHTO pullout friction factor (F*) equal to tanδ = tan(28.7°) = 0.55 (i.e., F* = tanδ = R_i_tanϕ = 0.7tan38° = 0.55).

^c^Dilatancy angle assumed as ψ = ϕ_s_ – 30°.

^d^Interface dilatancy angle assumed as equal to fill-soil dilatancy angle.

**Figure 9:**
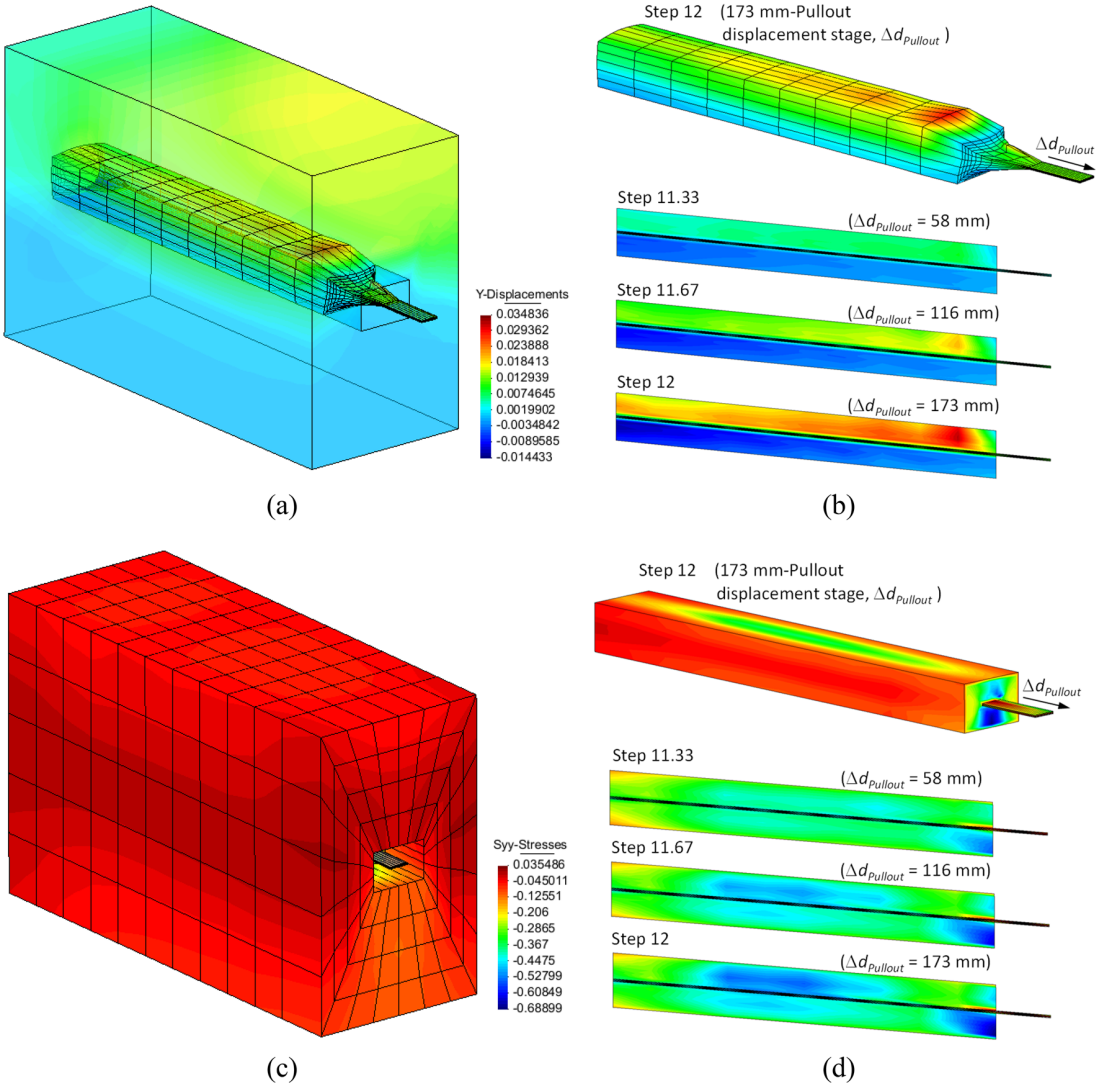
Base case vertical displacements (m) for (**a**) entire model, and (**b**) at interface with their evolution in time (i.e., steps) on interface vertical cross-length-section, and vertical stress (MPa) for (**c**) entire model, and (**d**) at interface with their evolution in time (i.e., steps) on interface vertical cross-length-section.

Figure 9 shows the vertical displacements and vertical stress evolution of the fill and interface materials during the pullout stage. Figure S1 of the Supplemental Material for this paper shows the shear and vertical stresses due to head displacement evolution along the reinforcement length. Positive-upwards displacements are generated during the pullout due to soil dilatancy (Fig. 9a). Displacements are more noticeable within the interface zone, due to greater shear strains, and near the frontal opening sleeve attributed to the prescribed boundary condition (Fig. 9b). The soil constitutive model uses a fixed value for dilatancy, whereas soils reach a critical dilatant state in which further shear deformations will occur without volume changes. Still, due to the range of displacements assumed in the model, realistic dilatancy effects are expected. Initial vertical stress values tended to zero near the frontal opening where the sleeve was modeled. Significant increments in vertical and shear stress are generated during pullout (Fig. 9c, Fig. S1) throughout almost all the reinforcement length. Vertical stress reaches values of 525 kPa, or approximately 10 times the assumed initial vertical stress, above the central zone (i.e., middle length) of the reinforcement strip, attributed to the effect of dilatancy (Fig. 9d). Maximum vertical stress location matches the with the highest shear stress zones (Fig. S1). Minor positive values (i.e., tensile stress) are observed within the later-side corner-boundaries, attributed to numerical equilibrium with the prescribed boundary conditions (i.e., prescribed normal displacements). Shear stresses show a symmetrical behaviour above and below the reinforcement (Fig. S1b).

**Figure 10:**
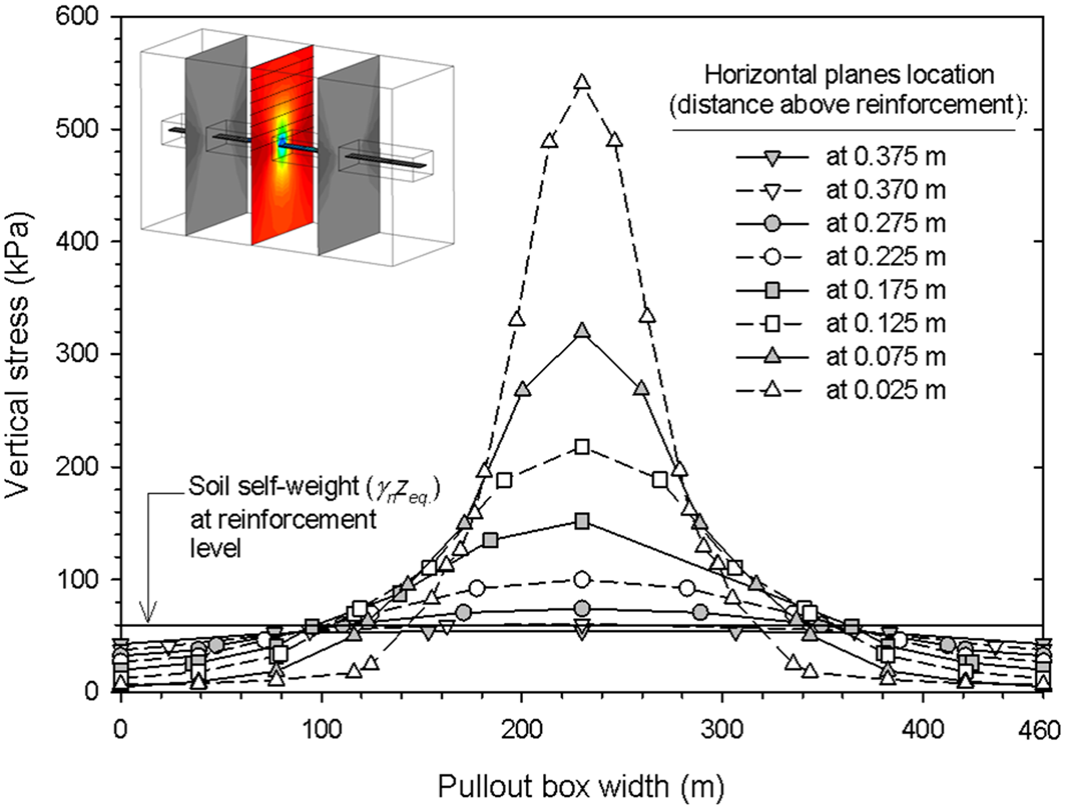
Vertical stress development at the interface in a vertical cross section planes 0.525 m from tail-end of reinforcement of the reinforcement layer at Step 12 (i.e., end of pullout test).

Figure 10 presents modelled vertical stresses at step 12 (i.e., after a complete pullout test) for several horizontal planes above the reinforcement within s vertical plane at 0.525 m from the tail-end of the reinforcement. Figure S2 shows results for vertical planes at 0.210 m (Fig. S2a) and 0.840 m (Fig. S2b) from the tail-end of the reinforcement. Modeled results show considerable variations between vertical pressure development at zones directly above the reinforcement and towards the lateral sides. This phenomenon is observable due to the 3D nature of the model. As previously observed (see Fig. 9d; Fig. S1a), vertical stress increments just above the reinforcement are generated due the shear strains caused by the pullout of the reinforcement as well as the effect of soil dilatancy, causing an increase of volume due to shear and, consequently upward displacement. Similar responses have been previously reported in pullout numerical models^37^. Stress distribution is several times higher than the pressure due to self-weight of the fill-soil above the reinforcement, while being several times lower towards the lateral side. Nevertheless, the equivalent resultant load in each and any horizontal plane remains constant as per the equivalent fill-soil depth due to soil-arching effect, even at the near-most plane towards the reinforcement. Results show that the considerable effect of dilatancy during pullout failure, as mentioned in the pioneering work of Lo^38^ and Alfaro and Pathak^39^. As expected, the magnitude of the vertical pressure at the central zone with regards to the vertical pressure distribution at the lateral sides is related to the horizontal plane location, where differences in vertical stress between central and lateral zones are accentuated closest to the reinforcement. Results tend to the corresponding surcharge load as the horizontal plane of analysis is further from the reinforcement.

### Sensitivity analysis

Sensitivity analyses were performed over various resisting parameters. Confining pressures scenarios remained unchanged. Table 2 details the analyzed parameter variations, including stiffness, friction and dilatancy angle for fill and interface soil, friction interaction factor, and reinforcement stiffness.

**Table 2:** Parameter variations for sensitivity cases.

Sensitivity on ^a^	Base case value	Variation cases	Case number
Soil friction angle, ϕ_s_ [°]	ϕ_s_ = 38 (R_i_ = 0.7; δ = 28.7)	Maintaining R_i_: ϕ_s_ = 32 (R_i_ = 0.7; δ = 23.6)	1
		ϕ_s_ = 44 (R_i_ = 0.7; δ = 34.1)	2
		Maintaining δ: ϕ_s_ = 32 (R_i_ = 0.88; δ = 28.7)	3
		ϕ_s_ = 44 (R_i_ = 0.57; δ = 28.7)	4
Soil and interface dilatancy^b^, ψ_s_ and ψ_i_ [°]	ψ_s_ = ψ_i_ = 8	ψ_s_ = ψ_i_ = 3	5
		ψ_s_ = ψ_i_ = 13	6
Soil-reinforcement friction interaction factor, f^’^ (and interface friction angle, δ) [–]	f^’^ = 0.55 (R_i_ = 0.7; δ = 28.7°)	f^’^ = 0.66 (R_i_ = 0.85; δ = 33.6°)	7
		f^’^ = 0.78 (R_i_ = 1; δ = 38°)	8
Soil (and interface) stiffness, E_s_ and E_i_ [MPa]	E_s_ = E_i_ = 20	E_s_ = 50 and E_i_ = 20	9
		E_s_ = E_i_ = 50	10
		E_s_ = 20 and E_i_ = 50	11
Reinforcement stiffness, E_r_ [MPa]	E_r_ = 210,000	E_r_ = 500	12

^a^Parameters no included here remain the same as in Base case.

^b^Despite the dilatancy definition (ψ = ϕ_s_ − 30°), the interface material dilatancy angle was assumed as equal to fill-soil dilatancy angle in Base case, and with the same value (i.e., ψ = 8°) for the complementary cases.

**Figure 11:**
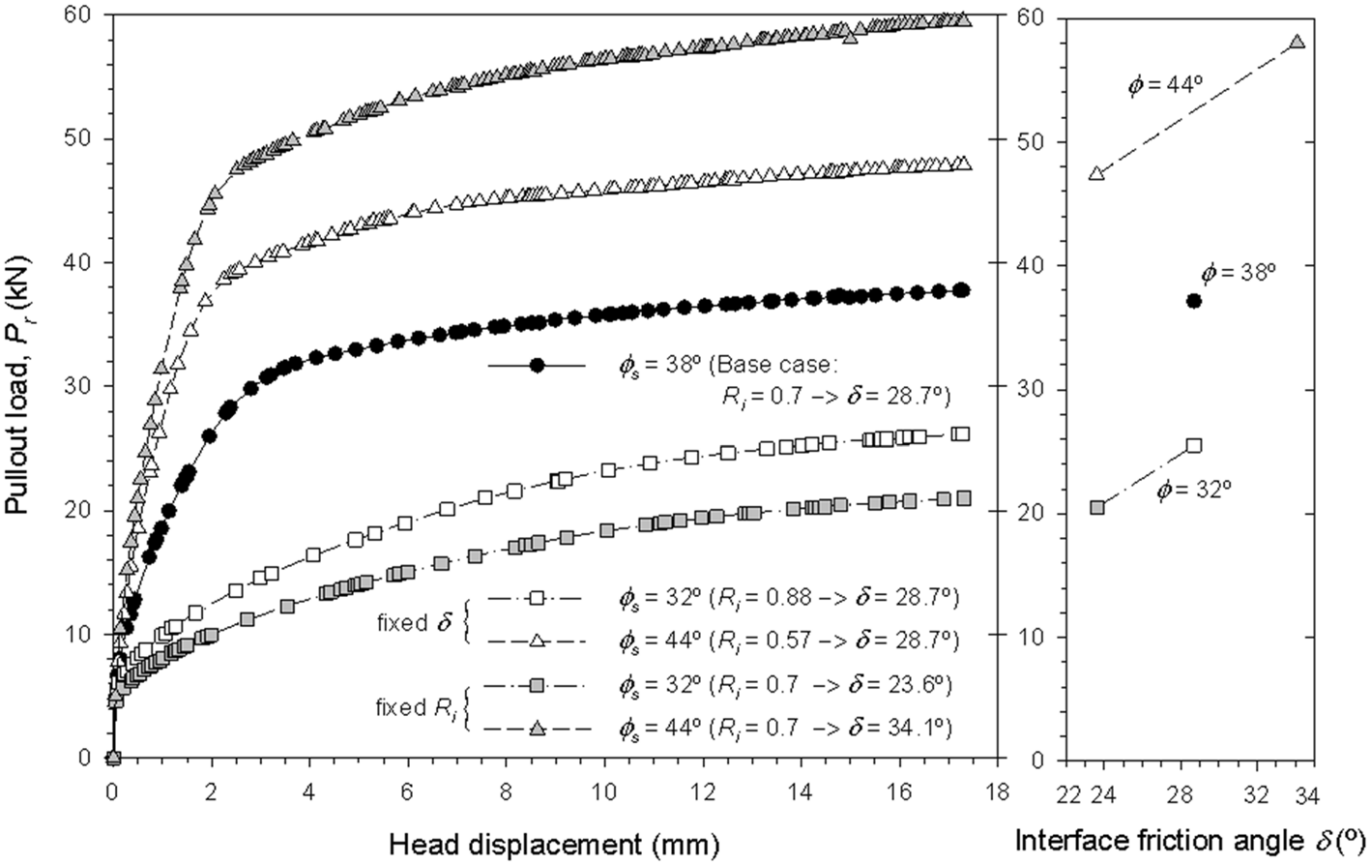
Axial reinforcement pullout load versus axial displacement response with regard to variations of soil friction angle (cases 1, 2, 3, and 4).

**Figure 12:**
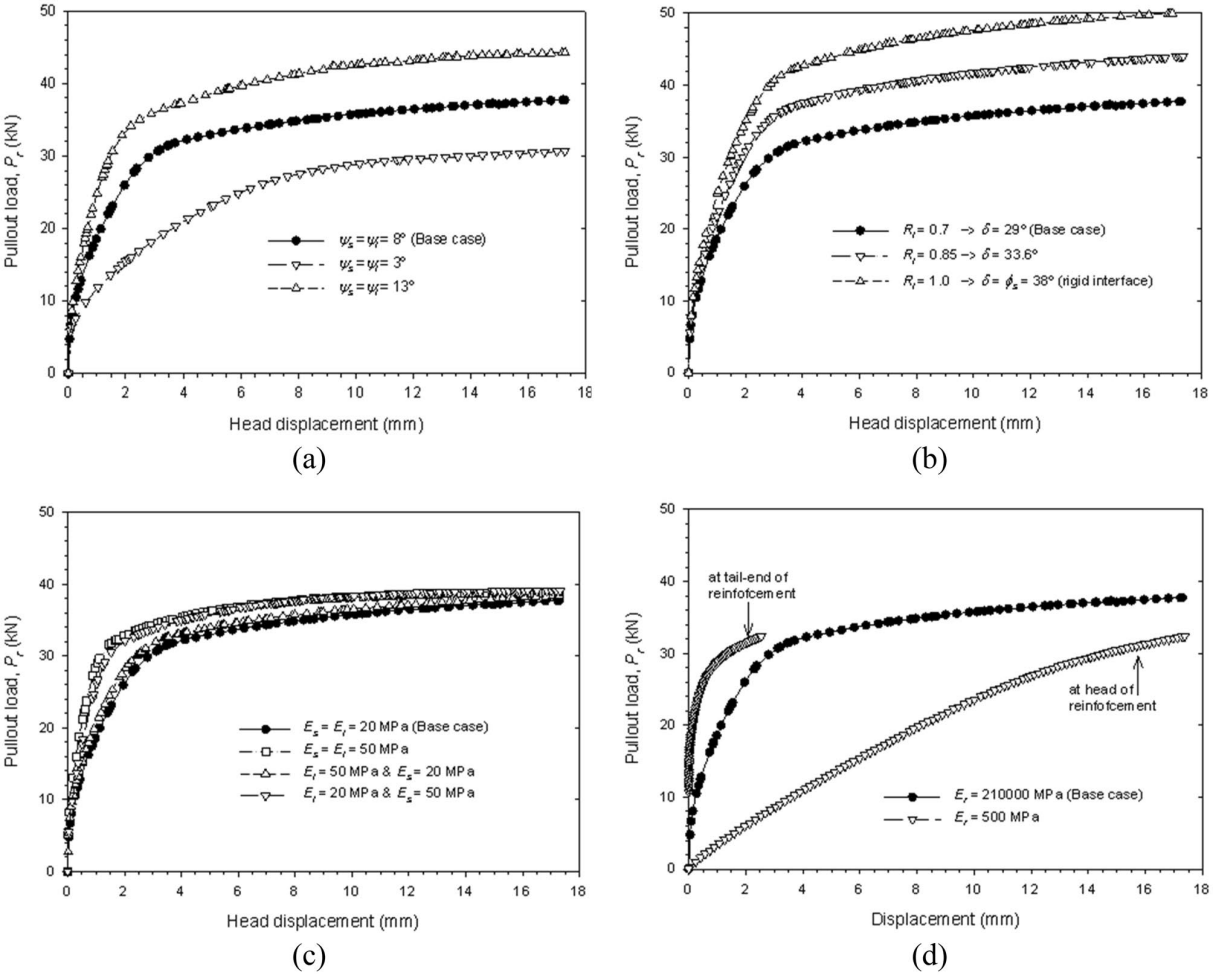
Axial pullout load versus axial displacement response with regards to variations on (**a**) dilatancy angle (cases 5 and 6), (**b**) interface reduction factor (cases 7 and 8), (**c**) stiffness (cases 9, 10, and 11), and (**d**) reinforcement stiffness (case 12).

Figure 11 shows the simulated pullout load at head of the reinforcement with respect to head-displacement for sensitivity cases 1, 2, 3, and 4. Analog results for the remaining cases are presented in Fig. 12. As strength increases (i.e., higher soil friction angle, ϕ), higher values of pullout load area obtained. A constant interface friction angles (δ) (i.e., cases 3 and 4), results in slighter variations than a constant interface strength reduction factor (R_i_) (i.e., cases 1 and 2) when compared to the base case. The interface friction angle appears to increase the pullout load for a constant soil-fill friction angle. Higher soil dilatancy (ψ) generates higher positive volumetric strains, resulting in increased vertical pressures, and, consequently, higher pullout load values (Fig. 12a). Likewise, for higher interface friction interaction factors (f^’^), which in turn results in higher internal friction angle values, higher pullout load values are obtained (Fig. 12b).

Higher stiffness (E) values result in more restrictions of vertical displacements due to dilatancy, thus, slightly higher pullout loads were observed (Fig. 12c). Results show that soil stiffness has more impact over pullout load than interface stiffness. For reinforcement elements, stiffness is related to the material extensibility. The base case represents an inextensible (e.g., steel) material, while the sensibility case represents an extensible (e.g., polymeric) material. An extensible reinforcement (case 12) reached pullout load values similar to those in the base case (i.e., critical load in which the elastic–plastic stress-regime is reached), but a considerably different displacement response. Due to the extensible nature of the material, displacements of the tail-end and head of the reinforcement are not the same (Fig. 12d).

Figure S3 shows the shear and vertical stress generation along the reinforcement length at step 12 for all sensitivity cases. Higher shear (Fig. S3a) and vertical (Fig. S3b) stresses are obtained for increased friction and dilatancy angles, for fill- and interface-soil. Increasing R_i_ yielded higher shear stresses but no significant variations in vertical stresses (Fig. S3c). For different soil stiffness values, no relevant variations in shear and vertical stress generation were observed (Fig. S3d). For different reinforcement stiffnesses, the somewhat constant shear stress distribution of the base case (i.e., inextensible strip) shifts to a variable distribution when simulating extensible reinforcement (case 12) with incrementing values from the back to the front (Fig. S3e). Likewise, vertical stresses change in distribution, increasing towards the reinforcement head due to the related soil and interface material dilatancy.

### Model calibration

After evaluating the base case response and sensitivity analysis results, the pullout model was calibrated for improved performance in specific scenarios with inextensible (i.e., steel ladder) and extensible (i.e., polymeric strip) reinforcements. Soil properties correspond to the tested sample for each pullout test (i.e., granular fill for steel ladders and low plasticity silty sand for polymeric strips). The calibration process was largely achieved through trial variations of the friction interaction factor.

#### Steel ladder reinforcement

For the inextensible reinforcement model, pullout test results for a steel ladder with 8 mm-diameter, 300 mm-separation transversal bars, and 1050 mm-length, 168 mm-width were used. Table 3 shows the reinforcement, fill- and interface-soil material parameters. Confining pressures included 0.375 m, 3.1 m, and 10.625 m of equivalent depth (z_eq_).

**Table 3:** Calibrated model parameters for the steel ladder pullout case.

Parameters	Materials					
	Steel ladder	Fill soil	Interface			
Unit weight (kN/m^3^)	75	20	20			
Elastic modulus, E (MPa)	84,446^a^	30^b^	30			
Poisson’s ratio, ν (–)	0.3	0.3	0.45^c^			
				Equivalent depths^d^		
				z = 0.4 m	z = 3 m	z = 10.5 m
Cohesion, c (kPa) ^e^	–	1		1	1	1
Friction angle, ϕ-soil and δ-interface (°)	–	40	Default^f^ (F* value)	28 (0.52)	22 (0.4)	15 (0.27)
			Calibrated (F* value)	89 (~ 60)	27 (0.5)	13 (0.23)
Dilatancy angle, ψ (°)^g^	–	10		10	10	10

^a^Equivalent stiffness from steel modulus (210 GPa) and actual steel ladder geometry (8 mm-diameter two longitudinal bars), converted to 50 mm-width × 5 mm-thick reinforcement 3D-model geometry.

^b^Value approximated from in-fill settlement reached after vertical surcharge, which is in agreement with soil material type (gravely sand) and performed compaction; as explained in previous sensitivity analysis (and demonstrated below for this current case) fill soil and interface stiffness variations implies different pullout-displacement trend, which can be properly refined to improve real pullout-displacement trend.

^c^Despite demonstrated to have no significant effect on pullout capacity, higher ν-value (i.e., ν = 0.45) was considered to best fit pullout capacity under high vertical pressure cases to mobilize confinement (i.e., to increase p-stress invariant) and reduce unrealistic volumetric interface plasticization.

^d^Equivalent depths from actual fill-soil layer height above the reinforcement and surcharge loading applied on top of pullout box.

^e^Non-zero cohesion value to ensure numerical stability at very low confining pressure.

^f^Default values from AASHTO^22^ pullout friction factor F* for steel grid reinforcement (bilinear value; see Fig. 8.13), i.e., from F* = 10(t/S_t_) = 10 × (8 mm/300 mm) = 0.27 (at depths z ≥ 6 m) linearly increasing up to F* = 20(t/S_t_) = 20 × (8 mm/300 mm) = 0.53 (at z = 0).

^g^Assumed as ψ =  ϕ − 30°.

**Figure 13:**
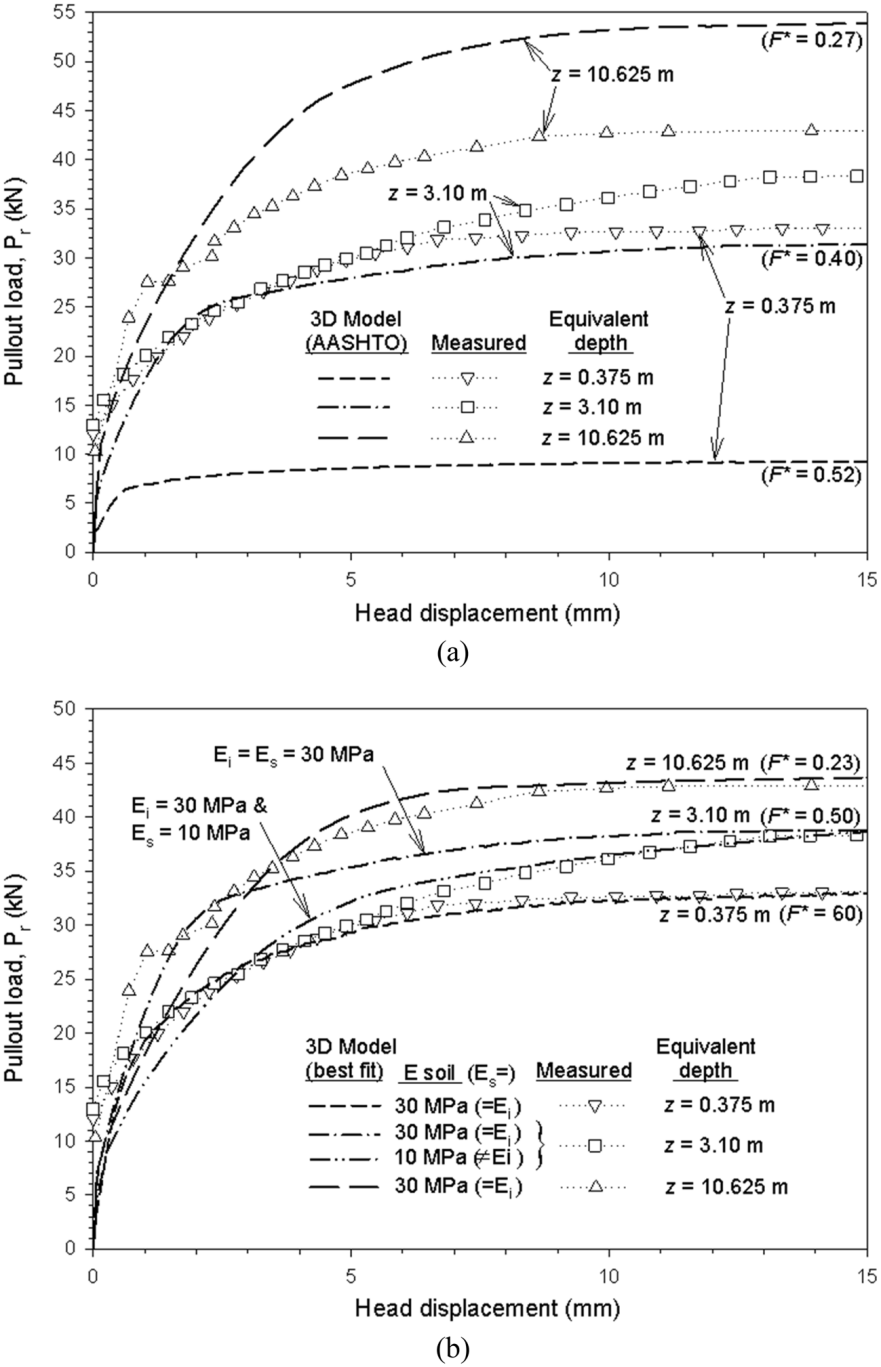
Head displacement and pullout load from measured and modeled steel ladder pullout tests results with (**a**) AASHTO (from F^*^ = 0.27 at z ≥ 6 m to 0.53 at z = 0), and (**b**) calibrated F^*^-values.

Figure 13 shows the head displacements versus pullout load results from the physical test and 3D model using steel ladder reinforcements. For F^*^ values based on AASHTO^22^ recommendations no proper agreement between measured and modelled results was obtained (Fig. 13a). At a lower confining pressure (z_eq_ = 0.375 m-depth) pullout load was underpredicted, while at a higher confining pressure (z_eq_ = 10.3625 m-depth) pullout load was overpredicted. An intermediate confining pressure scenario (z_eq_ = 3.10 m-depth) still presented inadequate results, but proved to have the best fit of the three load scenarios. Modifying F* values improved the modelled results at all depths (Fig. 13b). For an intermediate confining pressure (z_eq_ = 3.10 m-depth), slight modifications of soil stiffness allowed for a better fit. Figure S4 compares the measured, calibrated, and AASHTO-calculated friction interaction factor for various confining pressures. Reasonable agreement was obtained between calibrated and AASHTO-calculated values for intermediate and high confining pressures. For a low confining pressure, no agreement was obtained between F* values.

#### Polymeric strip reinforcement

For extensible reinforcements, pullout tests of a grade 70 (i.e., short-term strength of 70 kN), 90 mm-wide, polymeric strips were used for calibration. Table 4 shows the calibrated model parameters for reinforcement, fill- and interface-soil material. Confining pressures included 1-, 3.5-, and 7-m of equivalent depth. Test results show a similar response as those presented by Miyata et al.^12^, thus, while scarce, are deemed appropriate to be used in numerical models.

**Table 4:** Calibrated model parameters for the polymeric strip pullout case.

Parameters	Materials					
	Polymeric strip	Fill soil	Interface			
Unit weight (kN/m^3^)	75	21	21			
Elastic modulus, E (MPa)	500^a^	10^b^	10^b^			
Poisson’s ratio, ν (–)	0.3	0.3	0.45 ^c^			
				Equivalent depths^d^		
				z = 1.0 m	z = 3.5 m	z = 7.0 m
Cohesion, c (kPa)	–	14	Default^e^: Calibrated^f^:	9.4 1	9.4	9.4 14
Friction angle, ϕ-soil and δ-interface (°)	–	31	Default^g^ (F* value)	22 (0.4)	22 (0.4)	22 (0.4)
			Calibrated (F* value)	29 (0.56)	26.5 (0.5)	
Dilatancy angle, ψ (°)^h^	–	1		0	0	0

^a^Despite being a low-end value for polymeric strips^12^ after fixing other unknowns (as soil stiffness, see below), E_strip_ = 500 MPa demonstrated proper agreement with the measured pullout performance and related extensibility, in line with previous 3D numerical models for reinforced soil walls^35^.

^b^Value approximated from fill-soil settlement after applying vertical surcharge, in agreement with soil material type (silty sand) and performed compaction.

^c^Despite demonstrated to have not much significant effect on pullout capacity, higher ν_i_-value (i.e., ν_i_ = 0.45) was considered to best fit pullout capacity under high vertical pressure cases, to mobilize confinement (i.e., to increase mean stress p-invariant) and reduce unrealistic volumetric interface plasticization.

^d^Equivalent depths from actual fill-soil layer height above the reinforcement and surcharge loading applied on top of pullout box.

^e^Default interface cohesion value obtained from interface strength reduction (i.e., c_interface_ = R_i_ × c_soil_ = (tanδ/tanϕ_s_) c_soil_. = (tan(22°)/tan(31°)) c_soil_ = 0.67c_soil_. Thus, default c_interface_ value equal to 0.67 × 14 kPa = 9.4 kPa.

^f^Calibrated cohesion values as obtained from direct shear point-by-point data (i.e., less cohesion at low confining pressure, but also greater friction angle at that location).

^g^Default values from AASHTO^22^ pullout friction factor F* for geotextile and geogrid reinforcement type (δ = tan^−1^(F*) = tan^−1^(C_i_ × tanϕ) = tan^−1^ (0.67tan(31°)) = tan^−1^(0.4) = 21.9°).

^h^ψ =  ϕ_s_ − 30° = 1; however, interface material assumed as non-dilatant (i.e., ψ = 0).

**Figure 14:**
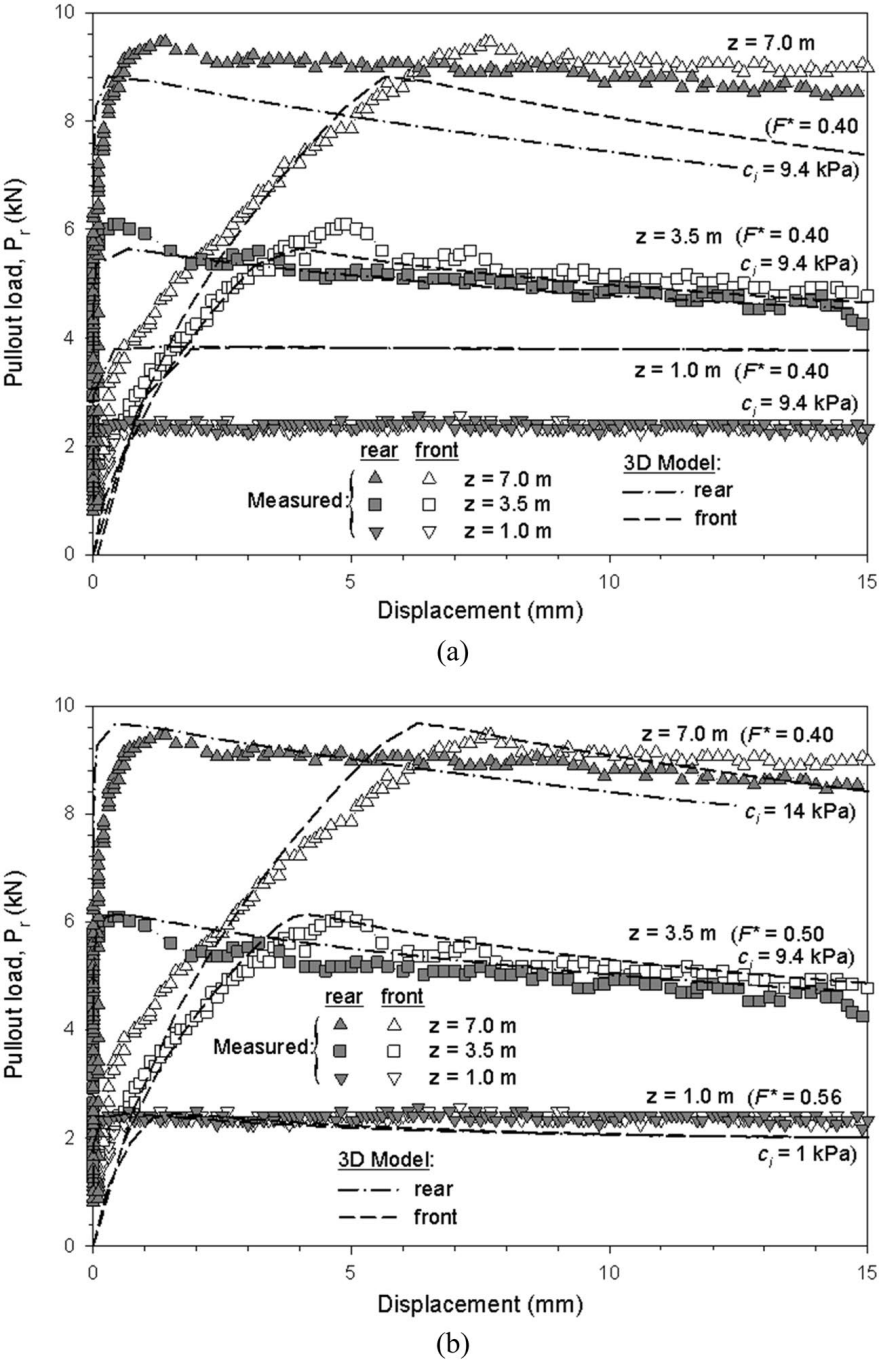
Displacements and pullout load results from measured and modeled polymeric strip pullout tests results for (**a**) AASHTO default value (F^*^ = 0.4), and (**b**) calibrated F^*^ and interface cohesion (c_i_) values.

**Figure 15:**
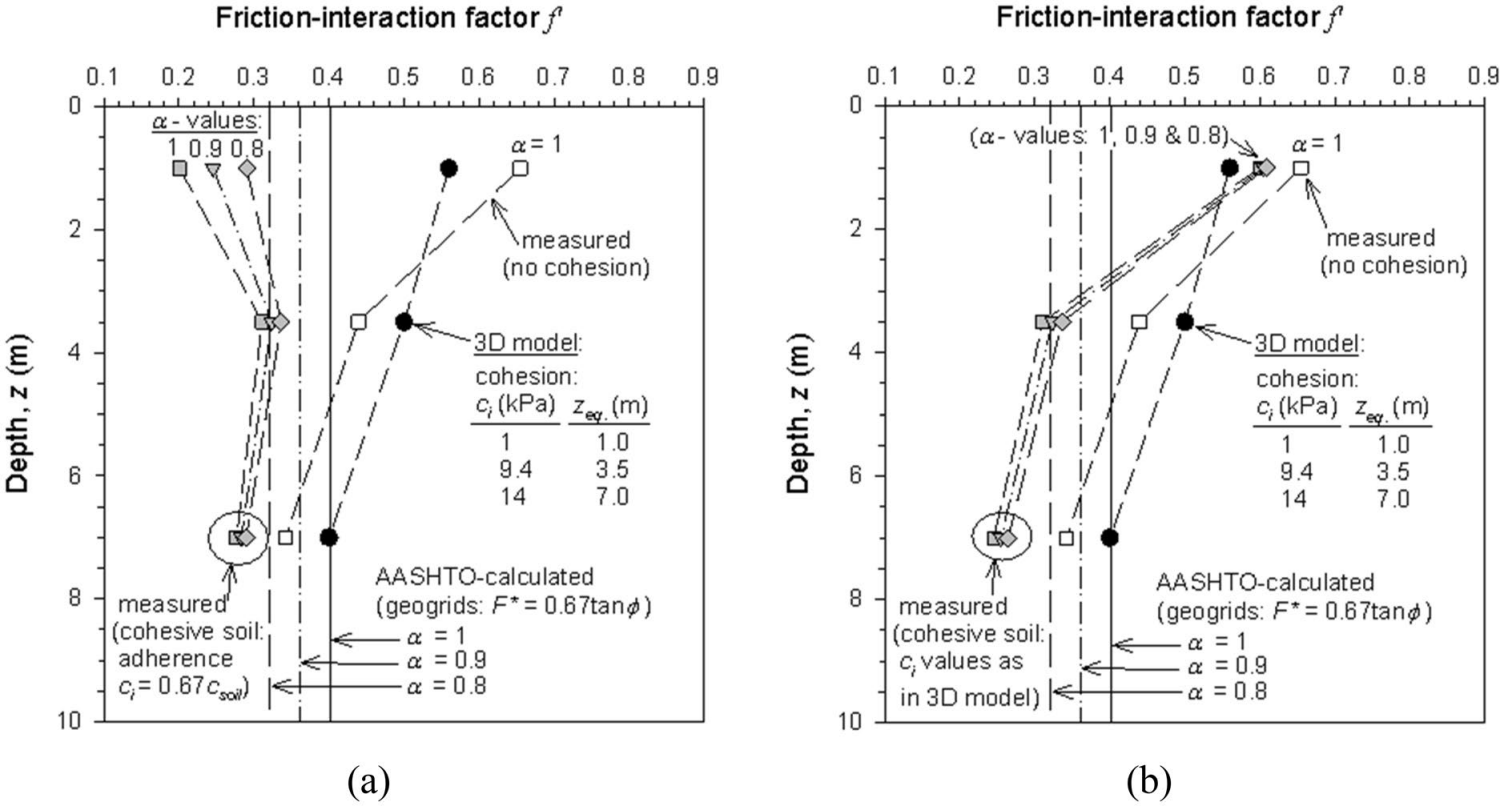
Comparison of calculated, measured and model-calibrated friction interaction factor (f^’^ = αF^*^) for polymeric strips pullout tests with and without cohesion values for (**a**) fixed interface adherence (c_i_ = 0.67c_s_), and (**b**) variable interface adherence as obtained in the calibrated 3D model.

Figure 14 shows displacements versus pullout loads results obtained from the physical tests and 3D model using polymeric reinforcements. Before calibration, moderate agreement was reached between measured and modelled results for all but the low confining pressure case (z_eq_ = 1 m-depth) (Fig. 14a). The first approach (non-calibrated) considered a fixed interface strength reduction for friction angle and cohesion (R_i_ = 0.67). By refining the pullout friction factor (F*) and the apparent interface cohesion values (c_i_), improved agreement was obtained between measured and modelled results at all simulated depths (Fig. 14b), including displacements at the head and tail-end of the reinforcement (Fig. S5). Parameter F^*^ was linearly decreased with depth while c_i_ was increased with depth (1 kPa at z_eq_ = 1 m-depth, 9.4 kPa at z_eq_ = 3.5 m-depth, and 14 kPa (c_i_ = c_s_) at z_eq_ = 7 m-depth). A variable friction interaction factor was required to improve model performance, in contrast to the constant value proposed in AASHTO^22^. Miyata et al.^12^ showed that a bi-linear model can predict, on average, conservative values, while showing no dependencies of predicted capacity with confinement pressures. Modeled results of f^’^ are in good agreement with measured values when considering a cohesionless soil (i.e., using Eq. 1), in which α = 0.9 shows a good fit for z_eq_ > 3.5 m. If soil cohesion is considered (i.e., using Eq. 4), a constant cohesion yields inadequate results at low confining pressures (i.e., z_eq_ = 1 m-depth) (Fig. 15a). When c_i_ increases with depth (i.e., using the model c_i_ values), results adjustment at low confining pressures improved while maintaining an adequate fit at middle and high confining pressures (Fig. 15b). Analog results were obtained when comparing the pullout friction factor (F*) of measured (with and without cohesion) and model values considering a fixed interface adherence (Fig. S7a) and a variable interface adherence (Fig. S7b).

The load–displacement simulations show a force decay response (see Fig. 14), which can be attributed to the extensible nature of the reinforcement. Relative displacements between front- and read-end provide an artificial fixture (as in, the reinforcement stretches progressively), which, after all the soil-reinforcement strength is mobilized, results in non-constitutive softening effect response.

As other 3D modelling attempts of polymeric reinforcements pullout tests have shown^15,16^, after a proper calibration, numerical models can represent the soil-reinforcement interface response under pullout load.

## Conclusions

The present work describes physical (i.e., in-lab) and 3D finite element models of pullout tests carried out using steel ladders and polymeric strips reinforcements at various equivalent depth scenarios. A base case 3D model was implemented as a first approach. Sensitivity analyses were performed using the base case model to identify the most relevant parameters influencing pullout results. The 3D model was then calibrated to better reproduce laboratory measured data for specific reinforcements (i.e., Steel ladders and polymeric strips). The main conclusion are as follows:

- Steel ladders pullout test results were in good agreement with literature-reported data. Physical results showed an overconservative estimation of pullout resistance following AASHTO^22^ guideless at low confining pressures, and proper agreement at increased depths. Values of soil-reinforcement interface friction angle (δ) of up to two times the soil friction angle were observed at low confining pressures.
- Polymeric strip pullout test results were in reasonable agreement with literature reported values. Pullout load results showed slight overconservative values at low confining pressures and slight overestimations with increased depths.
- Sensitivity analyses showed relevant dependencies of pullout load with soil friction and dilation angles, as well as interface reduction factor and reinforcement stiffness.
- After calibration, modeled results were in suitable agreement with measured values for all equivalent depths scenarios using steel ladder and polymeric strip reinforcements. Calibration was attained mainly by variations of the friction interaction factor (f’). For steel ladder cases, f’ was in reasonable agreement between model and AASHTO-calculated values for intermediate and high confining pressures, while no agreement was obtained at low confining pressures. For polymeric strips AASHTO^22^ guidelines define a fixed value. Contrary to this, the best representation of measured and modeled values was obtained with a variable friction interaction factor. The inclusion of cohesion in f’ only improved modeled and calculated values when a variable cohesion with depth was considered.
- The use of 3D numerical model allows for analyses of the in-plane stress distributions but require extensive computational effort, thus, are justified from an investigation point of view, but not necessarily for practical design.
- The agreement between physical and model results further validates the use of continuum elements for soil-reinforcement interfaces

The presented methodology may be of interest for designers when analyzing the soil-reinforcement pullout interaction, complementary to laboratory pullout testing. Before using numerical tools to predict pullout performance, modeled must be validated using proper laboratory or field measurements. Only after a proper calibration process, numerical results can be used to evaluated non-tested and complementary scenarios, such as soil or reinforcement properties variations.

### Supplementary Information

Supplementary Figures.

## Data Availability

Data is provided within the manuscript and supplementary information files.
