# Supplementary material for: Physical and 3D numerical modelling of reinforcements pullout test
Source: Sci Rep. 2024 Mar 28;14:7355. doi: 10.1038/s41598-024-57893-3 (PMC10978882; doi:10.1038/s41598-024-57893-3)
Supplement: Supplementary file 1 — Supplementary Figures. [file 41598_2024_57893_MOESM1_ESM.docx]

**Physical and 3D numerical modelling of reinforcements pullout test**

SUPPLEMENTARY MATERIAL

| 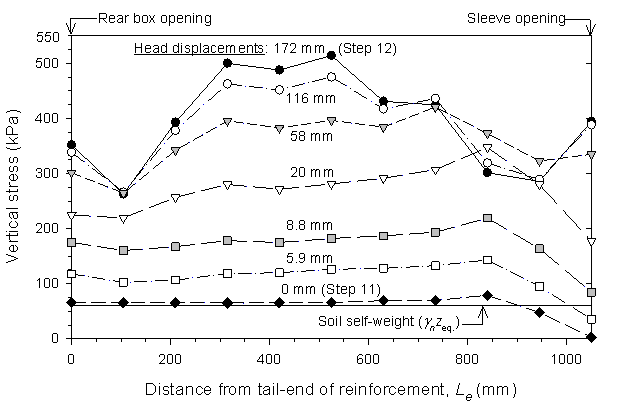 |
| --- |
| (a) |
| 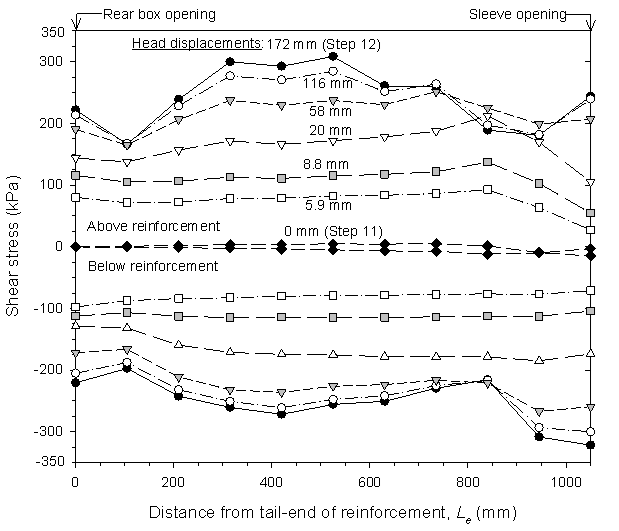 |
| (b) |

**Figure S1**. Evolution of (a) vertical and (b) shear stress due to reinforcement head displacement in the Base case pullout model.

| 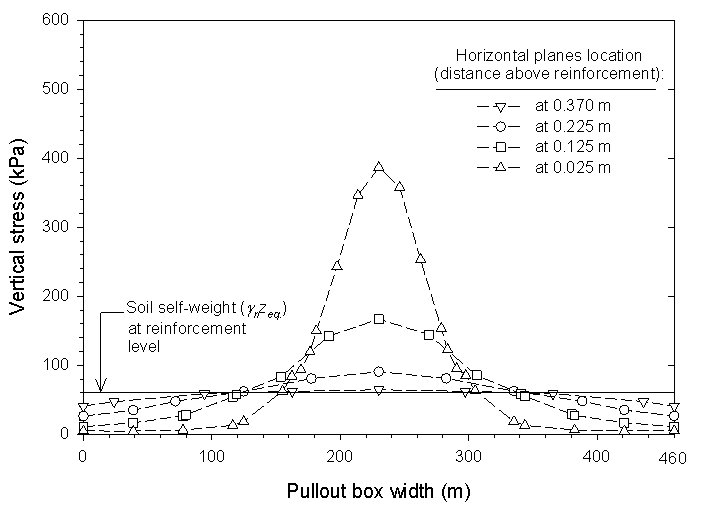 |
| --- |
| (a) |
| 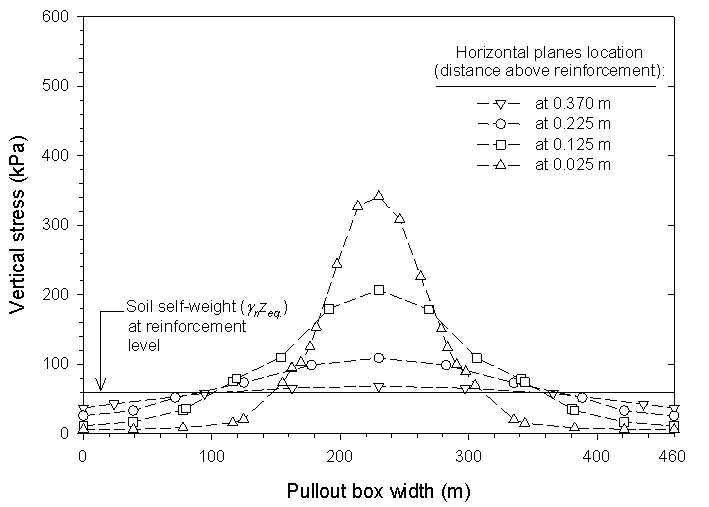 |
| (b) |

**Figure S2**. Vertical stress development of interface in a vertical cross section plane at (a) 0.210 m and (b) 0.840 m from tail-end of reinforcement at Step 12 (i.e., end of pullout test).

.

| 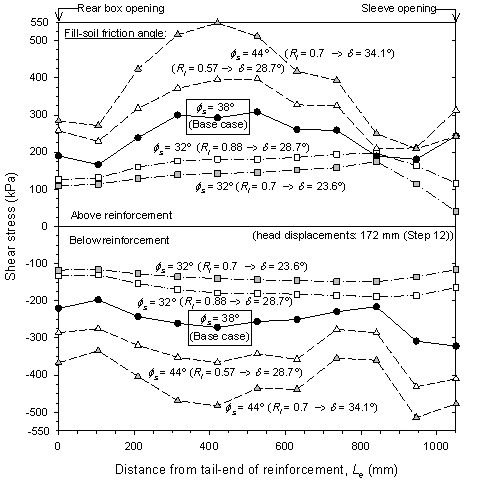 | 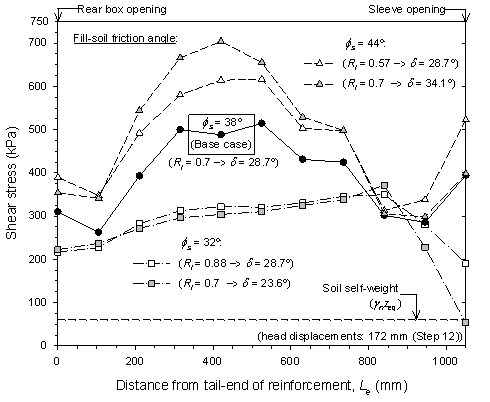 |
| --- | --- |
| (a) | |
| 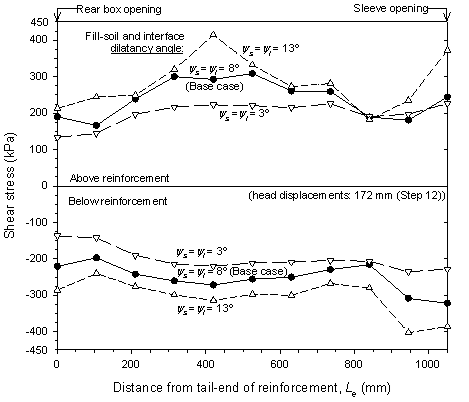 | 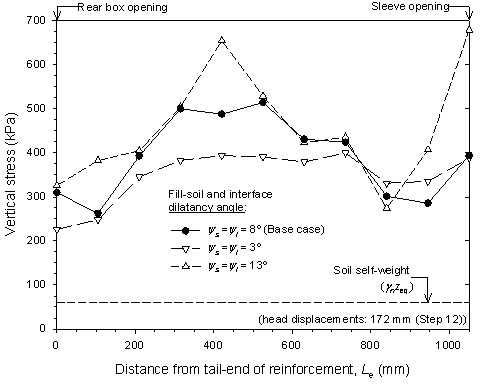 |
| (b) | |
| 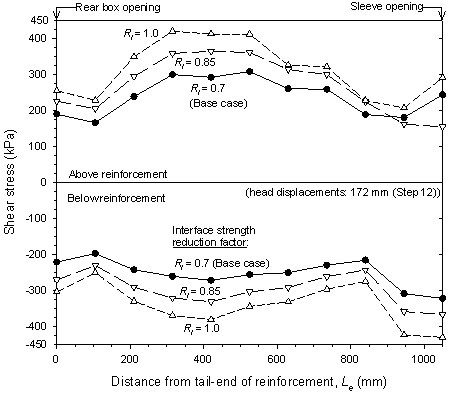 | 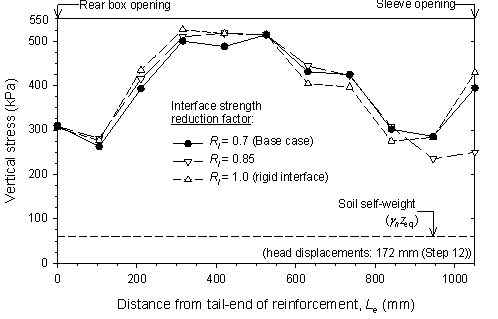 |
| (c) | |
| 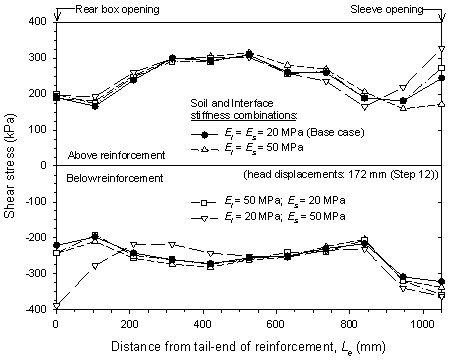 | 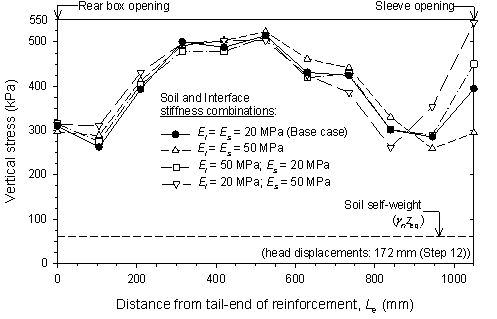 |
| (d) | |
| 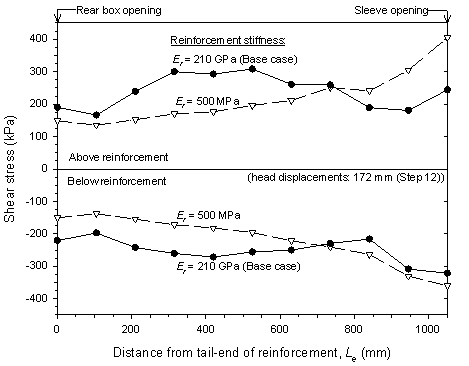 | 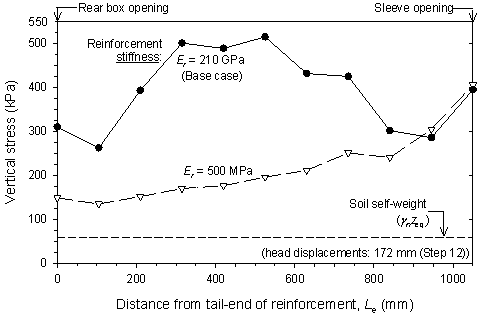 |
| (e) | |

**Figure S3**. Sensitivity results for shear (left) and vertical (right) stresses along the reinforcement length with variations on (a) soil friction angle (cases 1, 2, 3, and 4), (b) dilatancy angle (cases 5 and 6), (c) interface reduction factor (cases 7 and 8), (d) stiffness (cases 9, 10, and 11), and (e) reinforcement stiffness (case 12).

| 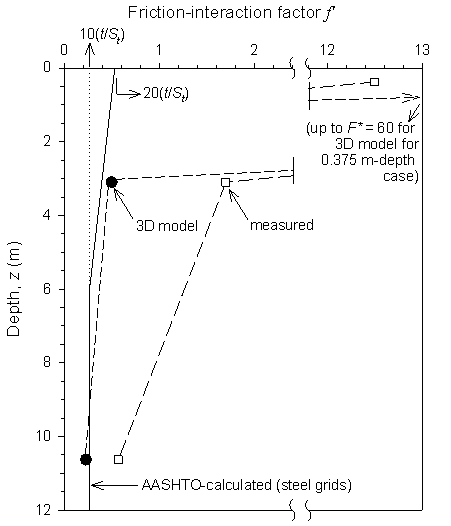 |
| --- |

**Figure S4**. Comparison of AASHTO-calculated, measured and 3D model-calibrated friction interaction factor (f^’^) for steel ladder pullout tests. *Note*: f^’^ = F^*^α = F^*^, as α = 1 for inextensible reinforcement.

| 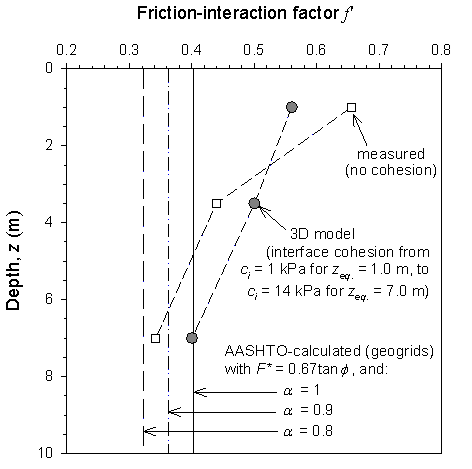 | 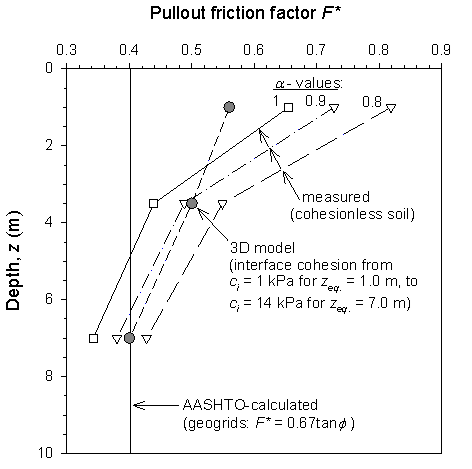 |
| --- | --- |
| (a) | (b) |

**Figure S5**. Comparison of calculated, measured and 3D model-calibrated (a) friction interaction factor (f^’^ = αF^*^), and (b) AASTHO-calculated pullout friction factor (F^*^) for polymeric strips pullout tests.

| 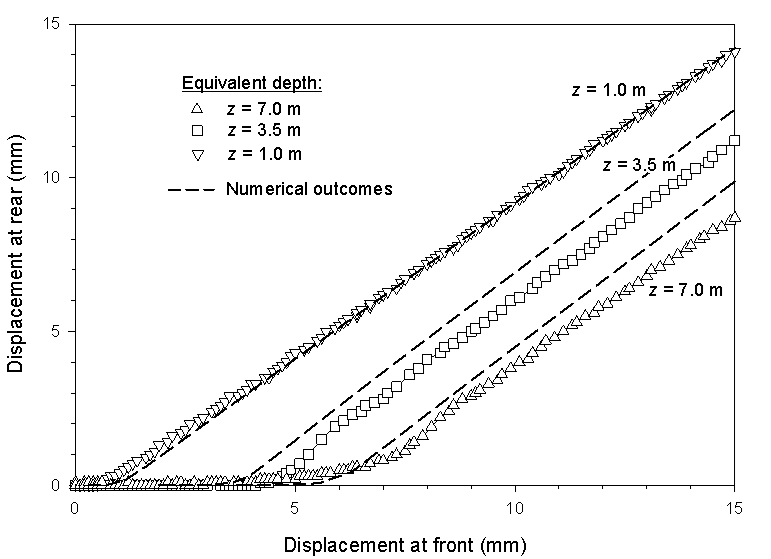 |
| --- |

**Figure S6**. Head and tail-end reinforcement displacements from measured and modeled polymeric strip pullout test results.

| 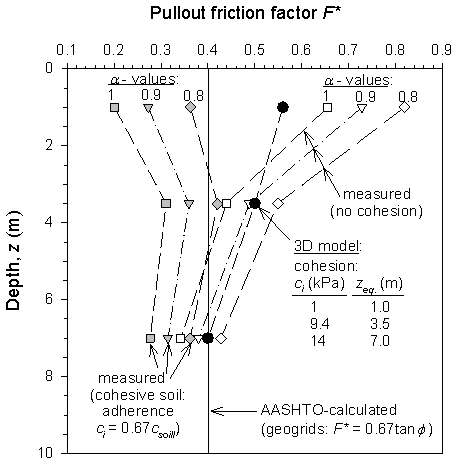 | 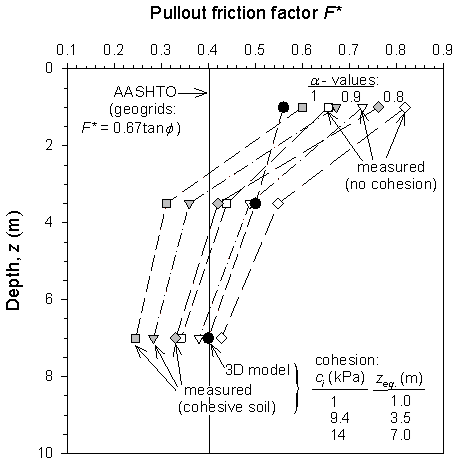 |
| --- | --- |
| (a) | (b) |

**Figure S7**. Comparison of calculated, measured and 3D model-calibrated AASHTO-calculated pullout friction factor (F^*^) for polymeric straps pullout tests with and without cohesion values for (a) fixed interface adherence (c_i_ = 0.67c_s_), and (b) variable interface adherence as obtained in best fit results from 3D model.

**END**
